# Supplementary material for: FHL2 Inhibits SARS-CoV-2 Replication by Enhancing IFN-β Expression through Regulating IRF-3
Source: Int J Mol Sci. 2023 Dec 26;25(1):353. doi: 10.3390/ijms25010353 (PMC10778585; doi:10.3390/ijms25010353)

Supplemental figures

FigS1:Original Western images used for preparing Figure.1E

Sample name:

Protein samples of Caco2 cells infected with 0.01moi SARS-CoV-2 at 12hpi, 24hpi and 36hpi

Target protein:

FHL2

MW:

32 KD

Lane: 4,5,6,7

Right to Left

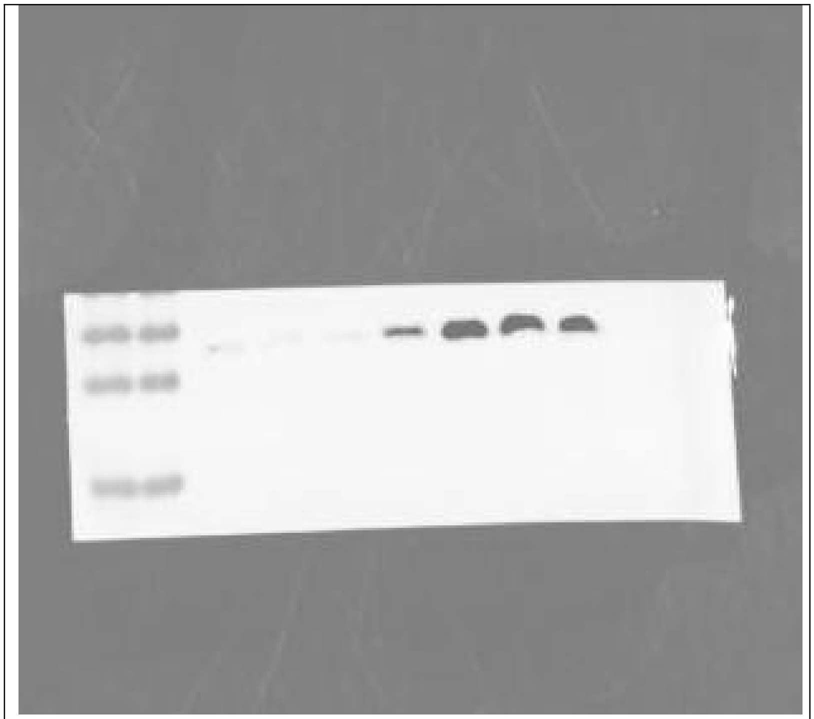

Sample name:

Protein samples of Caco2 cells infected with 0.01moi SARS-CoV-2 at 12hpi, 24hpi and 36hpi

Target protein:

α -tubulin

MW:

50 KD

Lane: 4,5,6,7

Right to Left

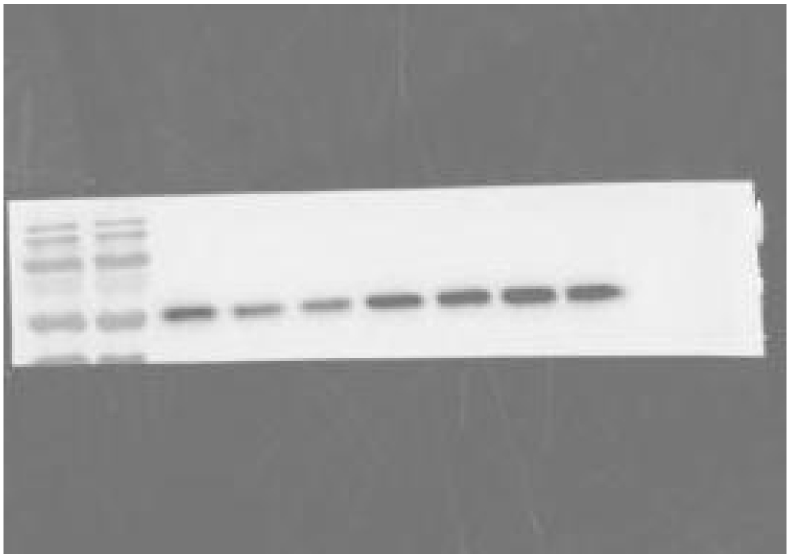

FigS2:Original Western images used for preparing Figure.1G

Sample name:

Protein samples of overexpressing FHL2 in Caco2 cells

Target protein:

N

MW:

55 KD

Lane: 2,3

Left to Right

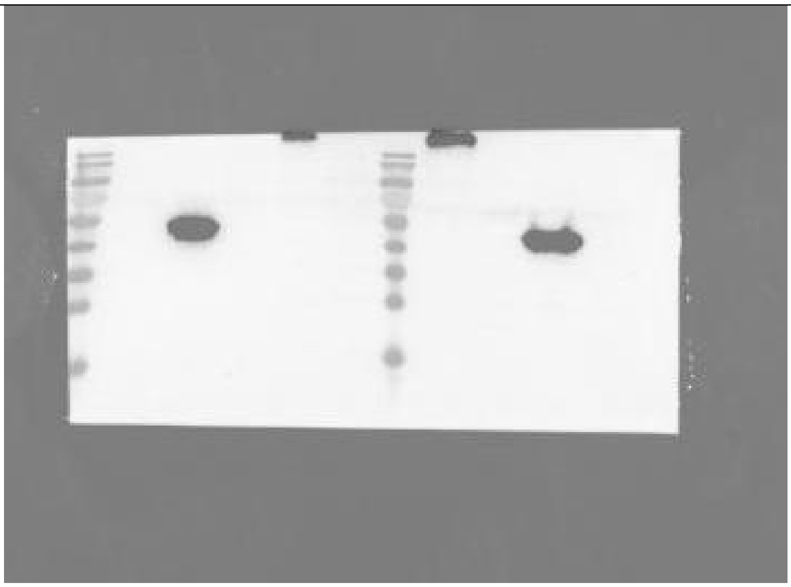

Sample name:

Protein samples of overexpressing FHL2 in Caco2 cells

Target protein:

FHL2

MW:

32 KD

Lane: 2,3

Left to Right

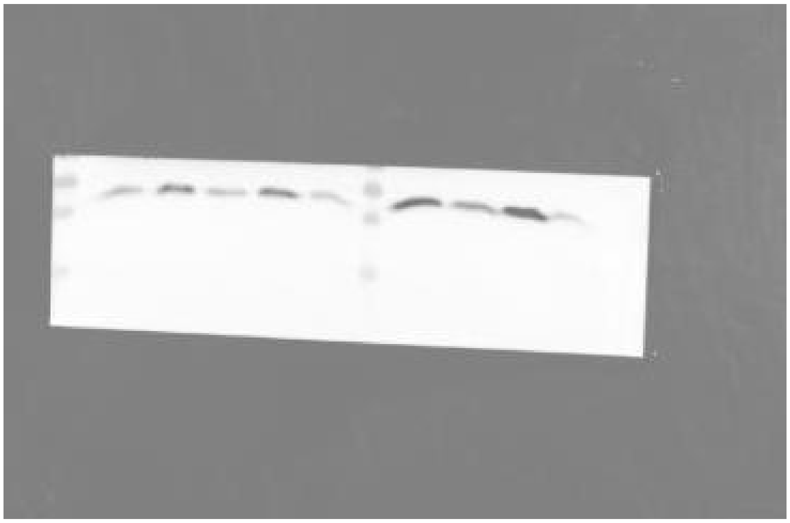

Sample name:

Protein samples of overexpressing FHL2 in Caco2 cells

Target protein:

α -tubulin

MW:

50 KD

Lane: 2,3

Left to Right

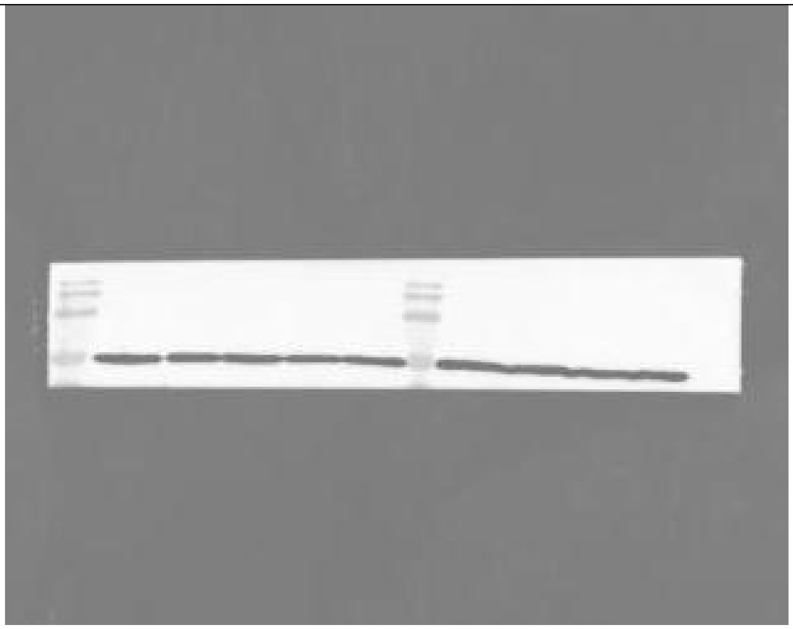

FigS3:Original Western images used for preparing Figure.2A

Sample name:

Protein samples of transfected with shRNA in Caco2 cells

Target protein:

FHL2

MW:

32 KD

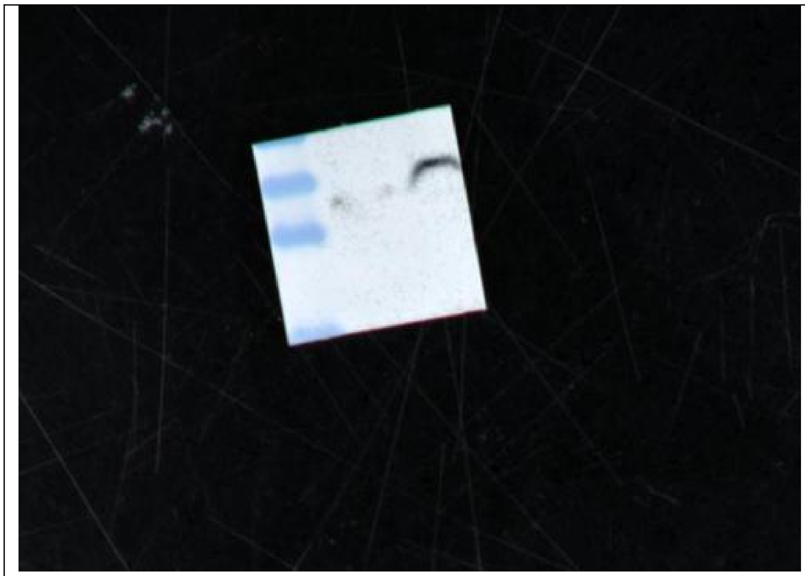

Sample name:

Protein samples of transfected with shRNA in Caco2 cells

Target protein:

α -tubulin

MW:

50 KD

Lane:1,2

Left to Right

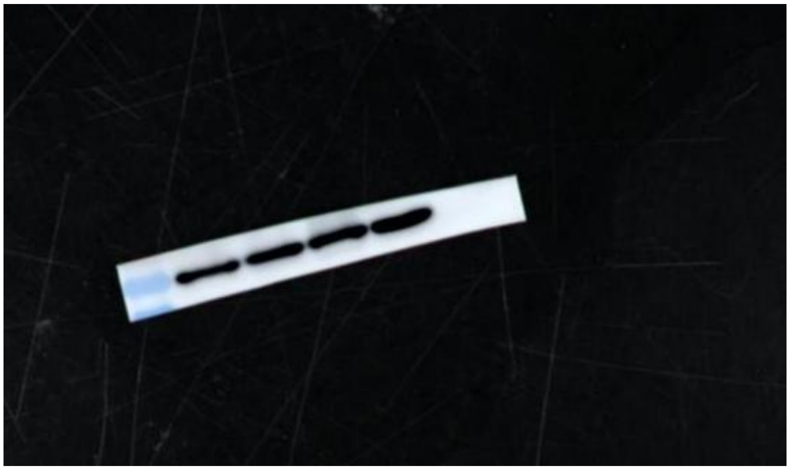

FigS4:Original Western images used for preparing Figure.2B

Sample name:

Protein samples of transfected with shRNA in 293T-ACE2 cells

Target protein:

FHL2

MW:

32 KD

Lane:1,2

Left to Right

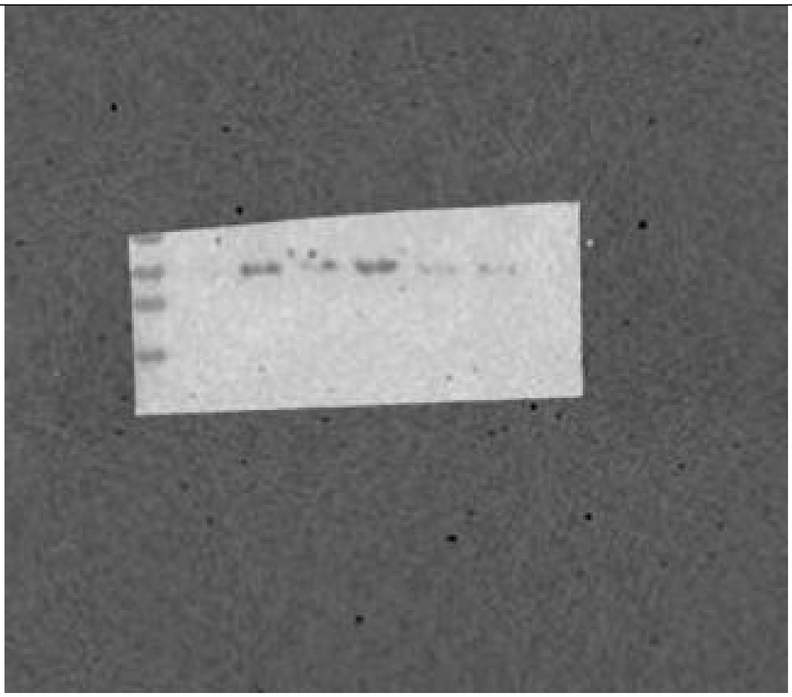

Sample name:

Protein samples of transfected with shRNA in 293T-ACE2 cells

Target protein:

α -tubulin

MW:

50 KD

Lane:1,2

Left to Right

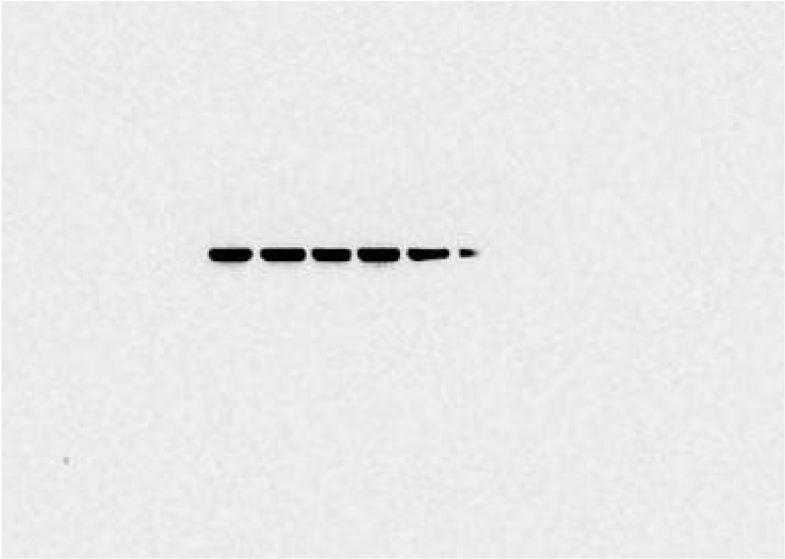

FigS5:Original Western images used for preparing Figure.2C

Sample name:

Protein samples of 0.01 MOI WT strain were infected in FHL2 knockdown Caco2 cells for 12 hours

Target protein:

N

MW:

55 KD

Lane: 5,6

Left to Right

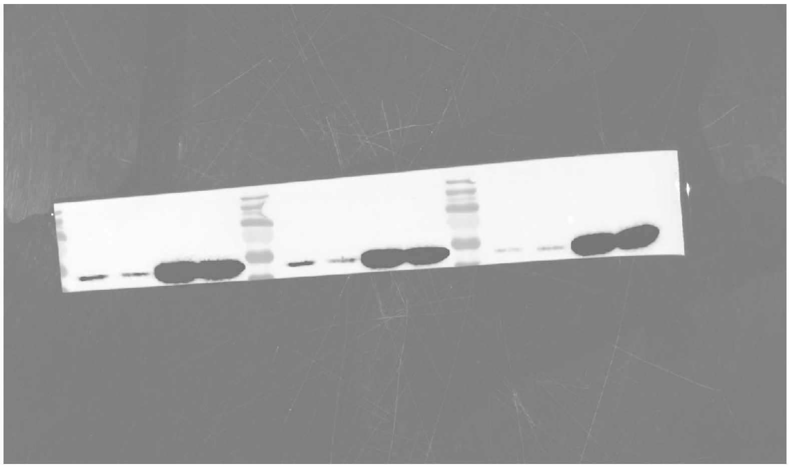

Sample name:

Protein samples of 0.01 MOI WT strain were infected in FHL2 knockdown Caco2 cells for 24 hours

Target protein:

N

MW:

55 KD

Lane: 7,8

Left to Right

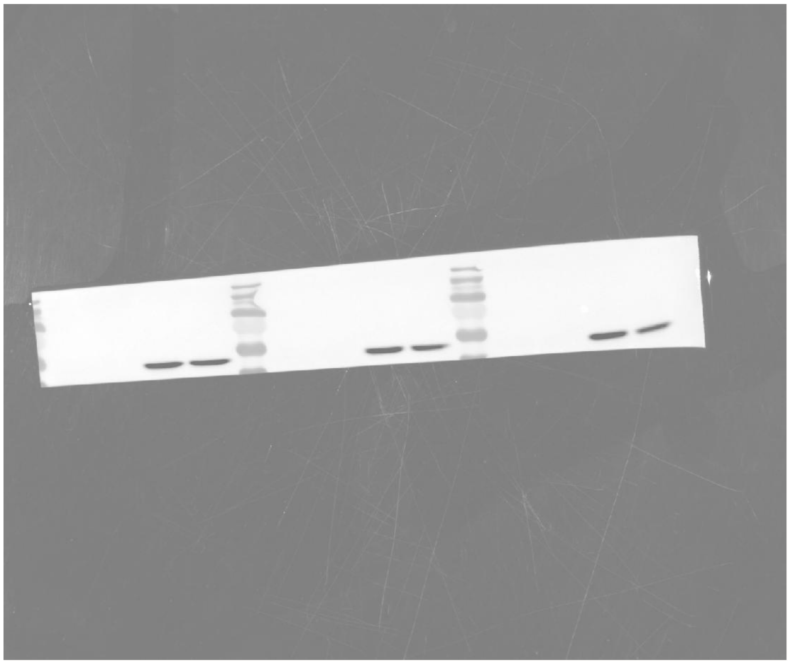

Sample name:

Protein samples of 0.01 MOI WT strain were infected in FHL2 knockdown Caco2 cells for 12 hours

Target protein:

GAPDH

MW:

37 KD

Lane: 5,6

Left to Right

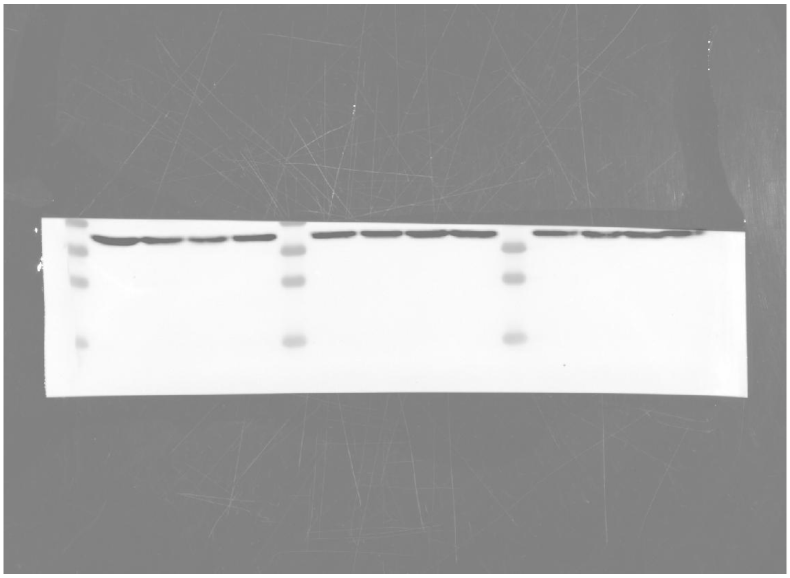

Sample name:

Protein samples of 0.01 MOI WT strain were infected in FHL2 knockdown Caco2 cells for 24 hours

Caco2 cells

Target protein:

GAPDH

MW:

37 KD

Lane: 7,,8

Left to Right

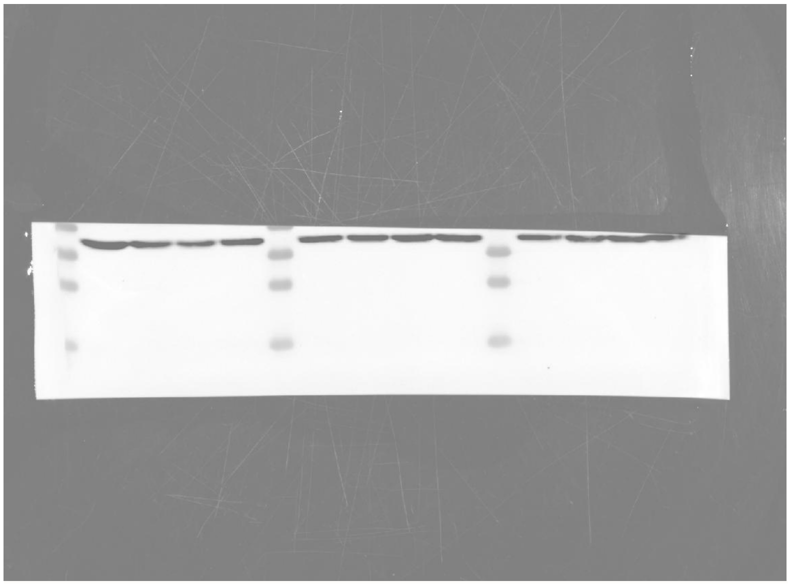

**Sample name:**<br>Protein samples of 0.1 MOI WT strain were infected in FHL2 knockdown Caco2 cells for 12 hours<br>**Target protein:**<br>N<br>**MW:**<br>55 KD<br><br>Lane: 1,2<br>Left to Right	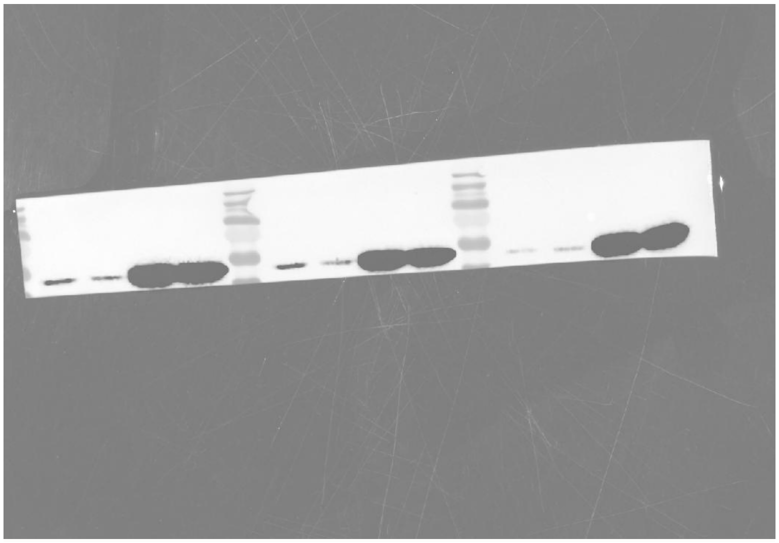
**Sample name:**<br>Protein samples of 0.1 MOI WT strain were infected in FHL2 knockdown Caco2 cells for 24 hours<br>Caco2 cells<br>**Target protein:**<br>N<br>**MW:**<br>55 KD<br><br>Lane: 3,4<br>Left to Right	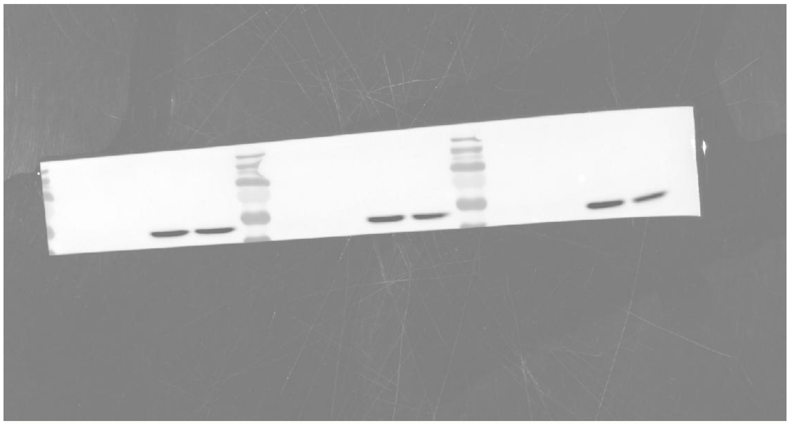

Sample name:

Protein samples of 0.1 MOI WT strain were infected in FHL2 knockdown Caco2 cells for 12 hours

Target protein:

GAPDH

MW:

37 KD

Lane: 1,2

Left to Right

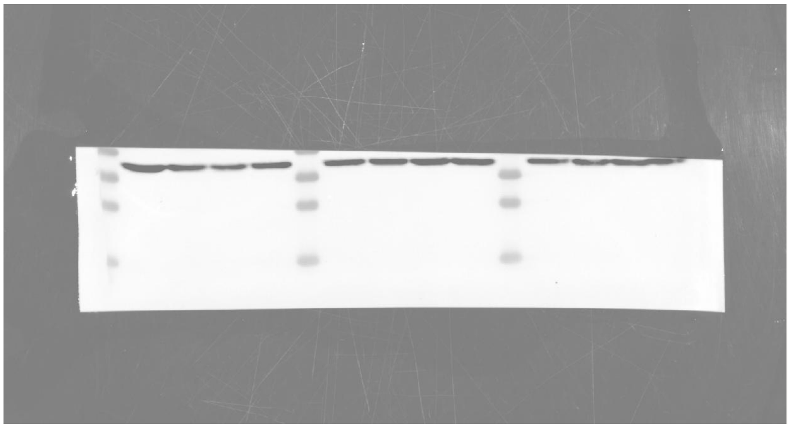

Sample name:

Protein samples of 0.1 MOI WT strain were infected in FHL2 knockdown Caco2 cells for 24 hours

Caco2 cells

Target protein:

GAPDH

MW:

37 KD

Lane: 3,4

Left to Right

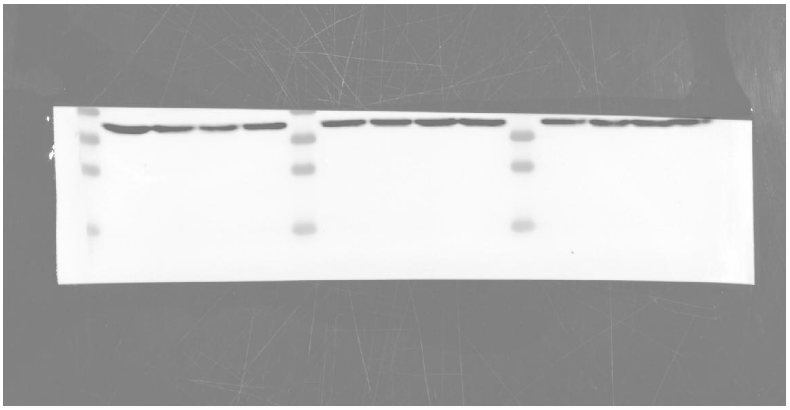

Sample name:

Protein samples of 0.01 MOI BA.1 strain were infected in FHL2 knockdown Caco2 cells for 12 hours

Target protein:

N

MW:

55 KD

Lane: 3,4

Left to Right

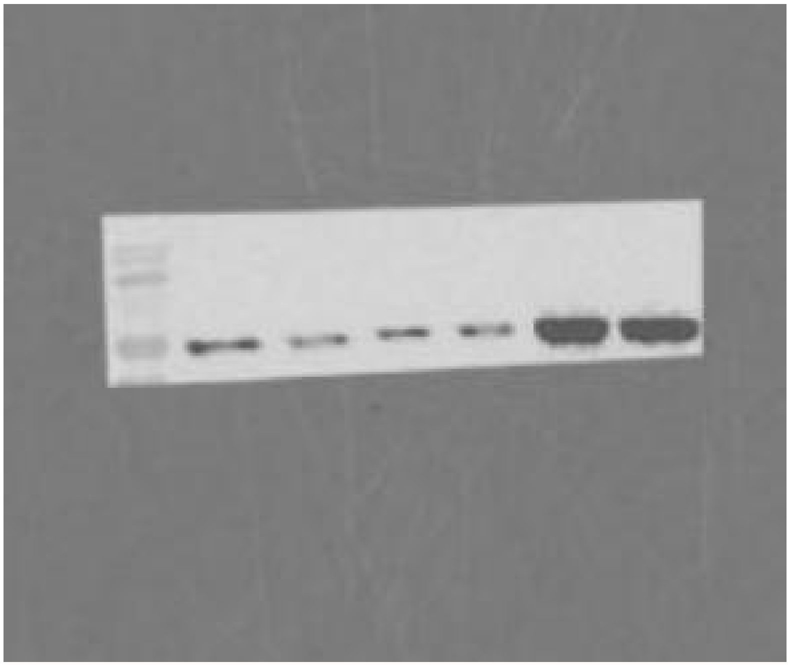

Sample name:

Protein samples of 0.01 MOI BA.1 strain were infected in FHL2 knockdown Caco2 cells for 24 hours

Target protein:

N

MW:

55 KD

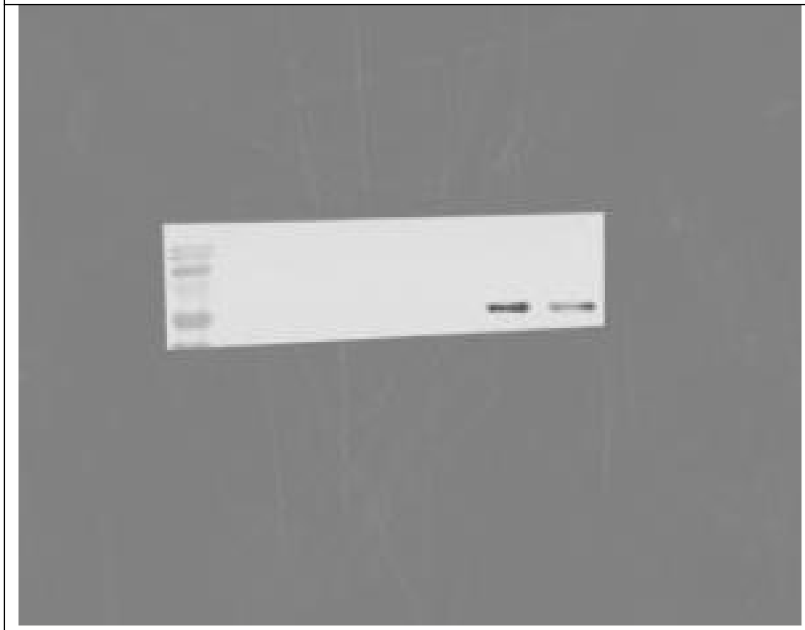

Sample name:

Protein samples of 0.01 MOI BA.1 strain were infected in FHL2 knockdown Caco2 cells for 12 hourss

Target protein:

GAPDH

MW:

37 KD

Lane: 3,4

Left to Right

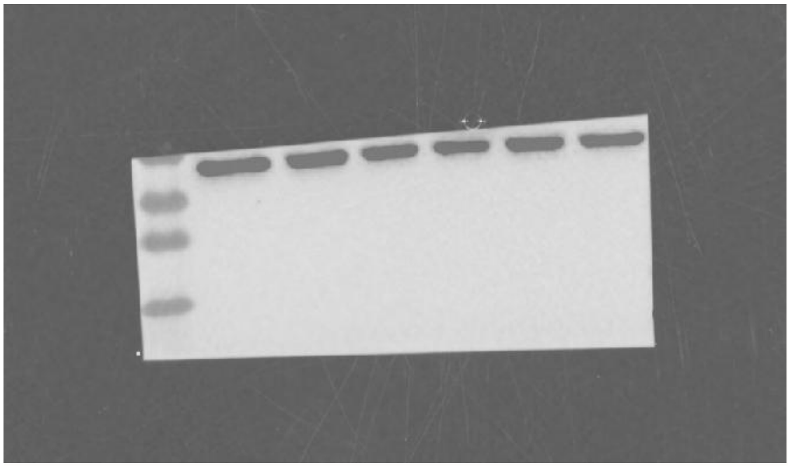

Sample name:

Protein samples of 0.01 MOI BA.1 strain were infected in FHL2 knockdown Caco2 cells for 24 hours

Target protein:

GAPDH

MW:

37 KD

Lane: 5,6

Left to Right

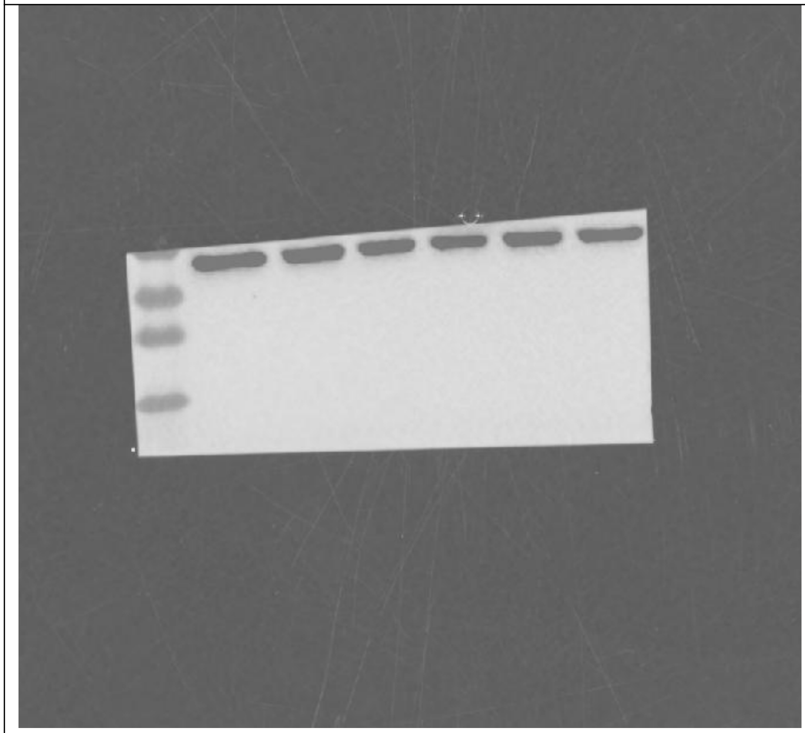

FigS6:Original Western images used for preparing Figure.2D

Sample name:

Protein samples of 0.01 MOI WT strain were infected in FHL2 knockdown 293T-ACE2 cells for 12 hours

Target protein:

N

MW:

55 KD

Lane: 3,4

Left to Right

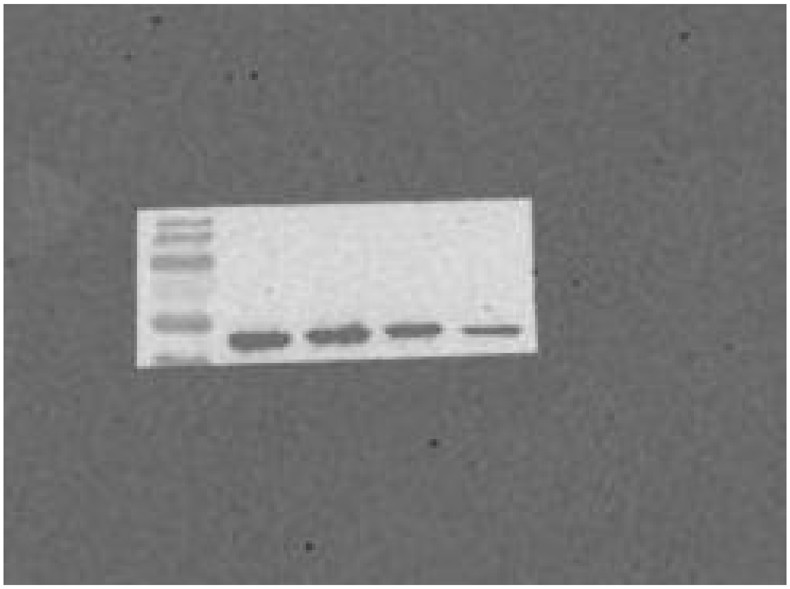

Sample name:

Protein samples of 0.01 MOI WT strain were infected in FHL2 knockdown 293T-ACE2 cells for 24 hours

Target protein:

N

MW:

55 KD

Lane: 1,2

Right to Left

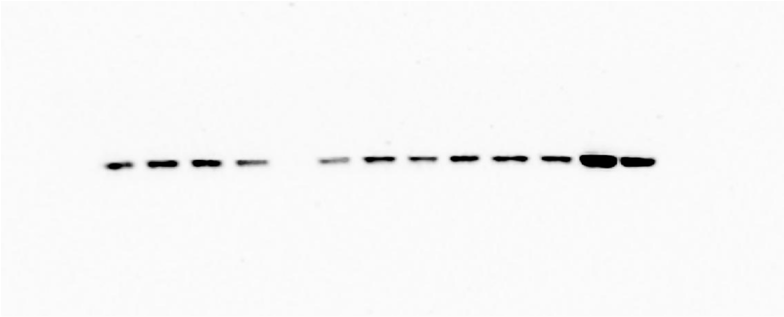

**Sample name:**<br>Protein samples of 0.01 MOI WT strain were infected in FHL2 knockdown 293T-ACE2 cells for 12 hours<br>**Target protein:**<br>GAPDH<br>**MW:**<br>37 KD<br><br>Lane: 3,4<br>Left to Right	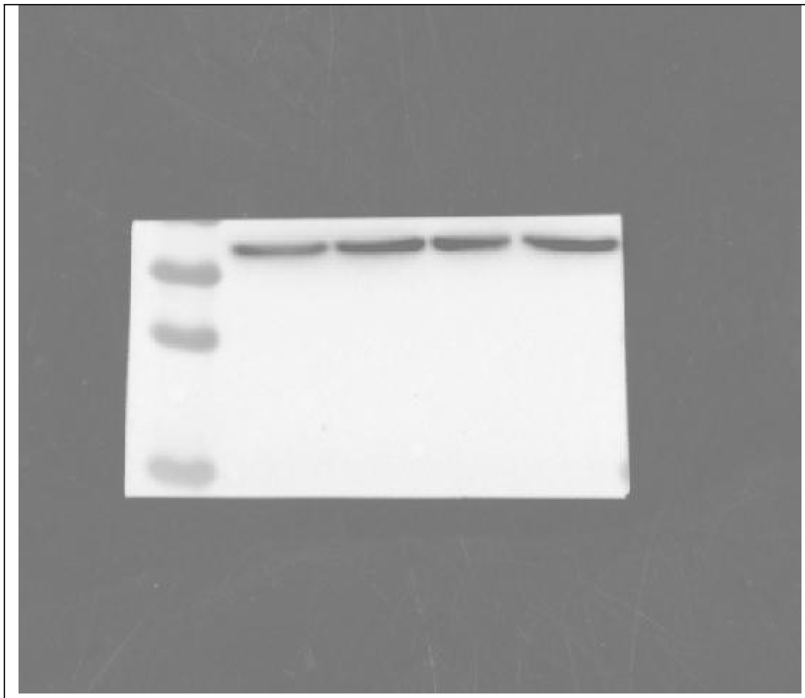
**Sample name:**<br>Protein samples of 0.01 MOI WT strain were infected in FHL2 knockdown 293T-ACE2 cells for 24 hours<br>**Target protein:**<br>GAPDH<br>**MW:**<br>37 KD<br><br>Lane: 1,2<br>Right to Left	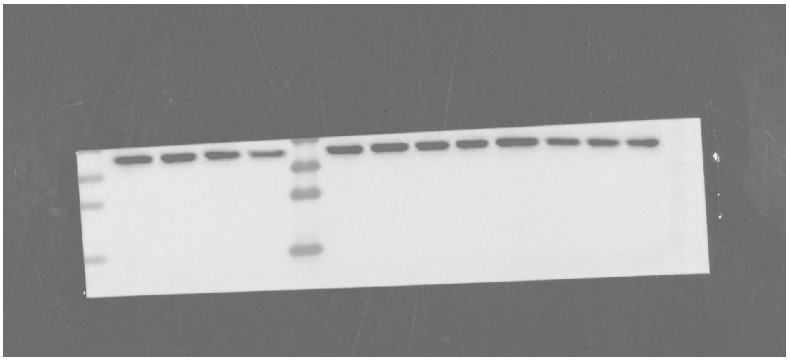

Sample name:

Protein samples of 0.01 MOI BA.1 strain were infected in FHL2 knockdown 293T-ACE2 cells for 12 hours

Target protein:

N

MW:

55 KD

Lane: 1,2

Left to Right

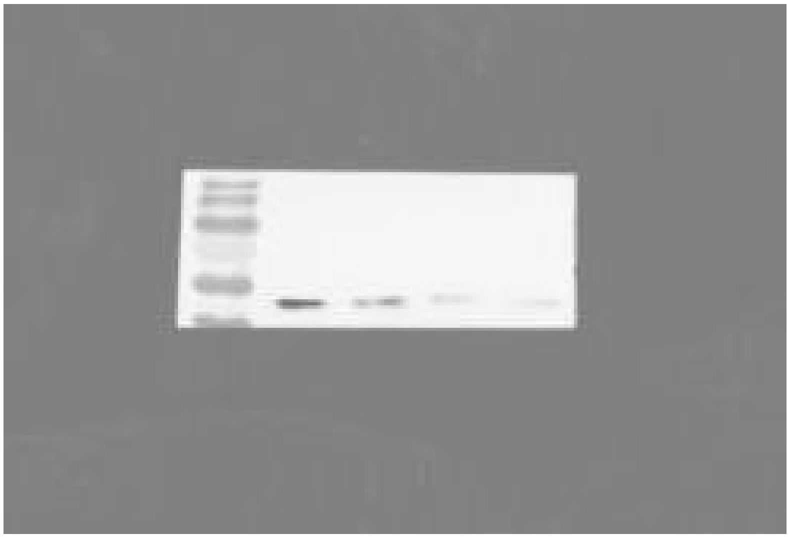

Sample name:

Protein samples of 0.01 MOI BA.1 strain were infected in FHL2 knockdown 293T-ACE2 cells for 24 hours

Target protein:

N

MW:

55 KD

Lane: 7,8

Left to Right

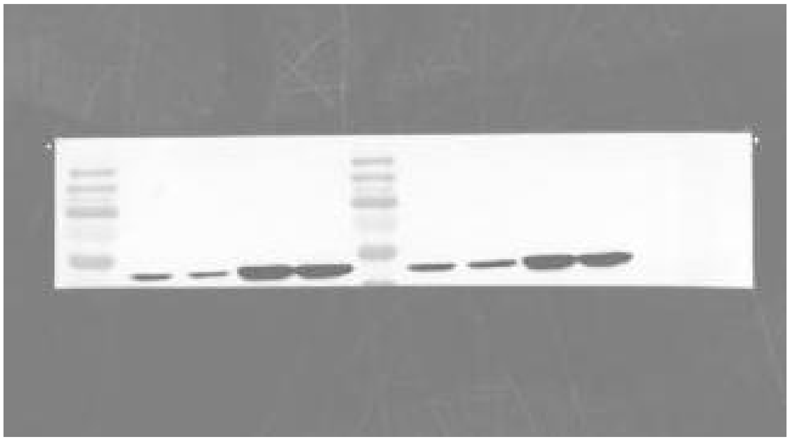

Sample name:

Protein samples of 0.01 MOI BA.1 strain were infected in FHL2 knockdown 293T-ACE2 cells for 12 hours

Target protein:

GAPDH

MW:

37 KD

Lane: 1,2

Left to Right

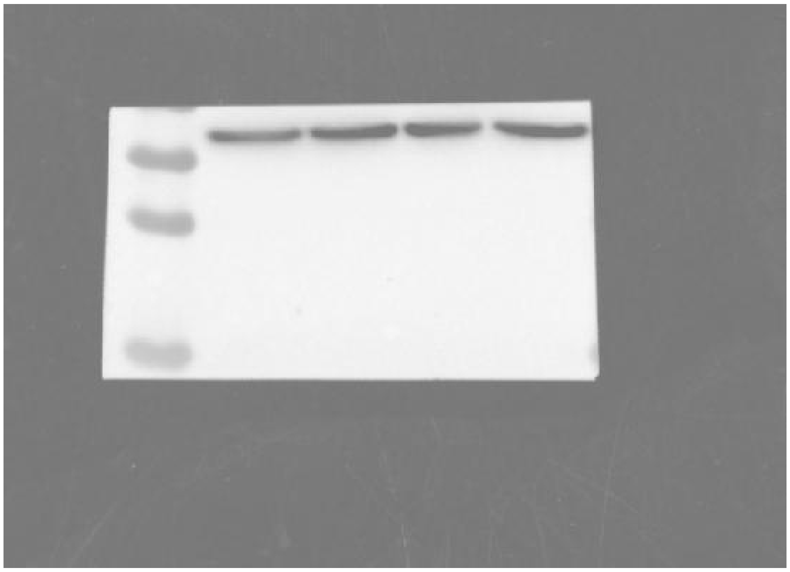

Sample name:

Protein samples of 0.01 MOI BA.1 strain were infected in FHL2 knockdown 293T-ACE2 cells for 24 hours

Target protein:

GAPDH

MW:

37 KD

Lane: 7,8

Left to Right

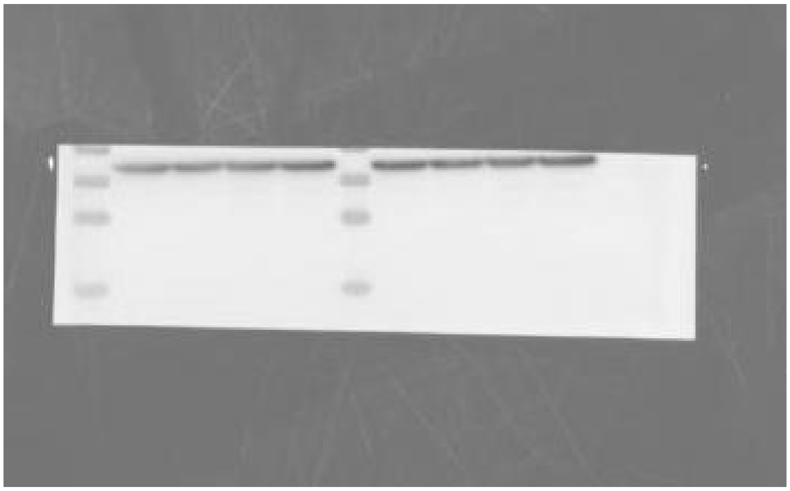

FigS7:Original Western images used for preparing Figure.3A

**Sample name:**<br>Protein samples of Caco2 cells transfected with FHL2-his eukaryotic expression plasmid<br>**Target protein:**<br>FHL2-his<br>**MW:**<br>32 KD<br><br>Lane: 5,6<br>Left to Right	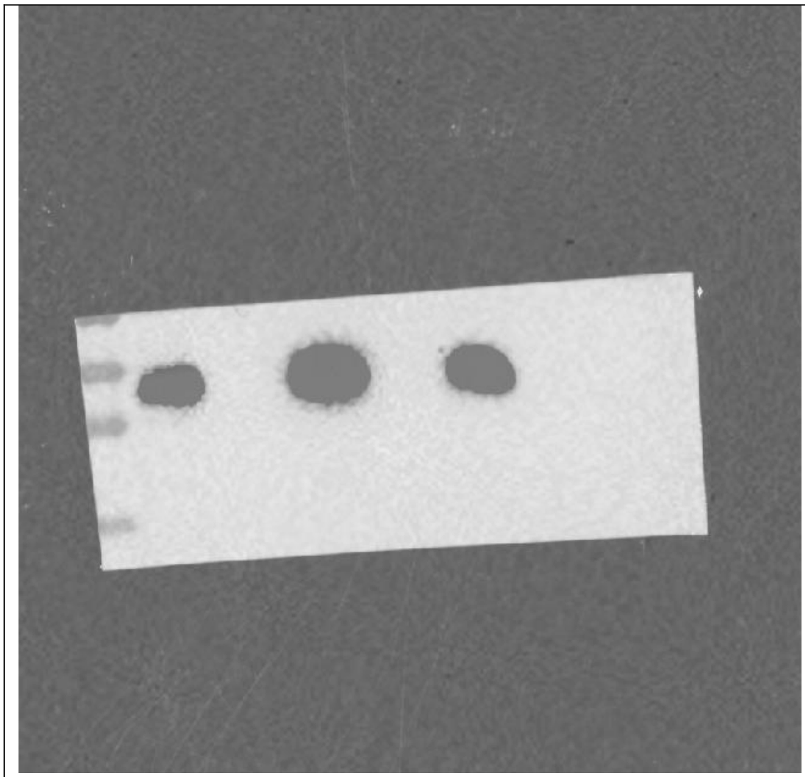
**Sample name:**<br>Protein samples of Caco2 cells transfected with FHL2-his eukaryotic expression plasmid<br>**Target protein:**<br>FHL2<br>**MW:**<br>32 KD<br><br>Lane: 5,6<br>Left to Right	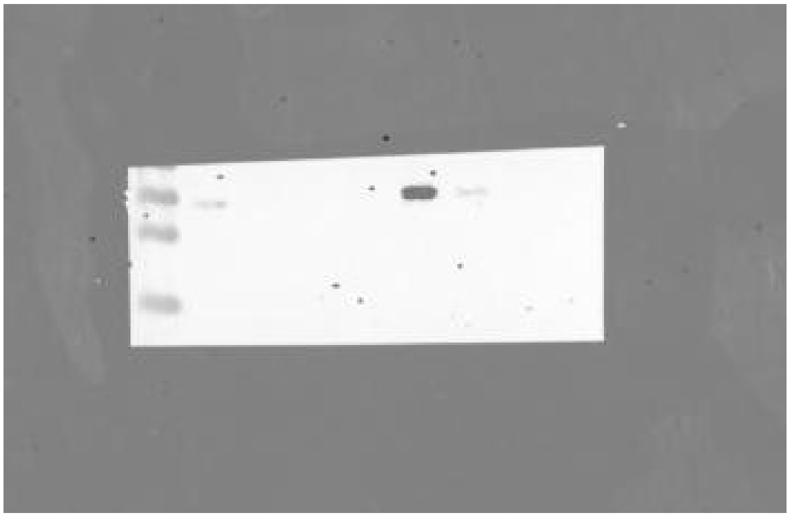

Sample name:

Protein samples of Caco2 cells transfected with FHL2-his eukaryotic expression plasmid

Target protein:

α -tubulin

MW:

50 KD

Second row

Lane: 5,6

Left to Right

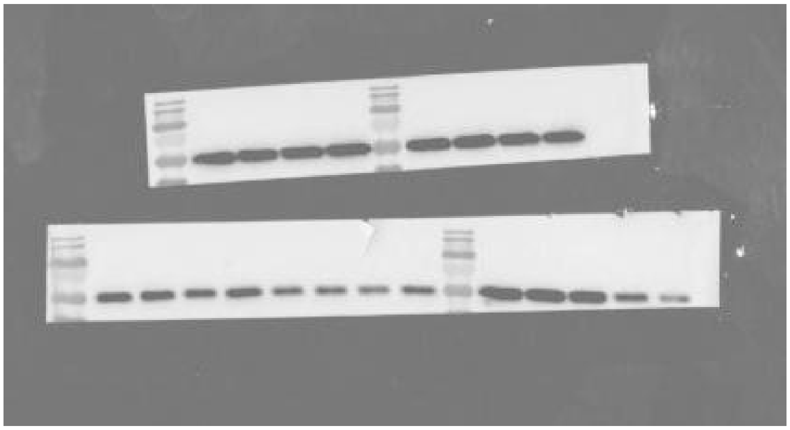

FigS8:Original Western images used for preparing Figure.3B

Sample name:

Protein samples of

293T-ACE2 cells transfected

with FHL2-his eukaryotic

expression plasmid

Target protein:

FHL2-his

MW:

32 KD

Lane: 1,2

Left to Right

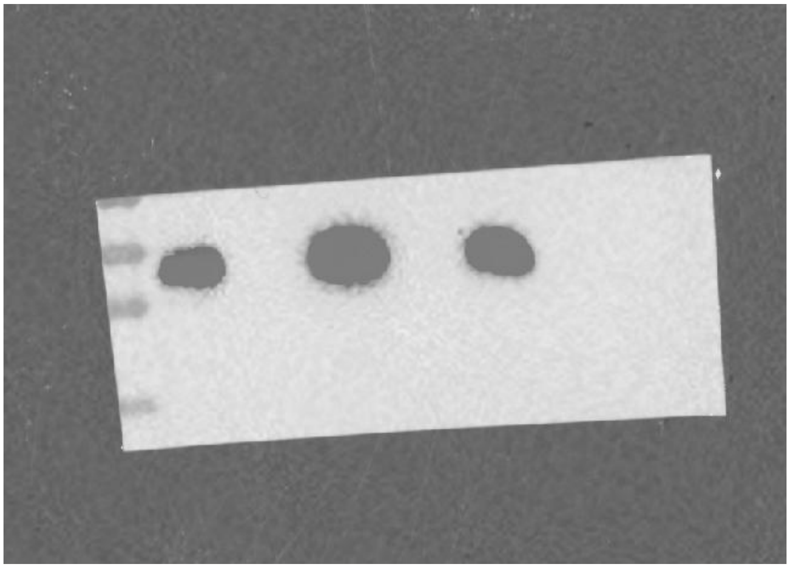

Sample name:

Protein samples of

293T-ACE2 cells transfected

with FHL2-his eukaryotic

expression plasmid

Target protein:

FHL2-his

MW:

32 KD

Lane: 3,4

Left to Right

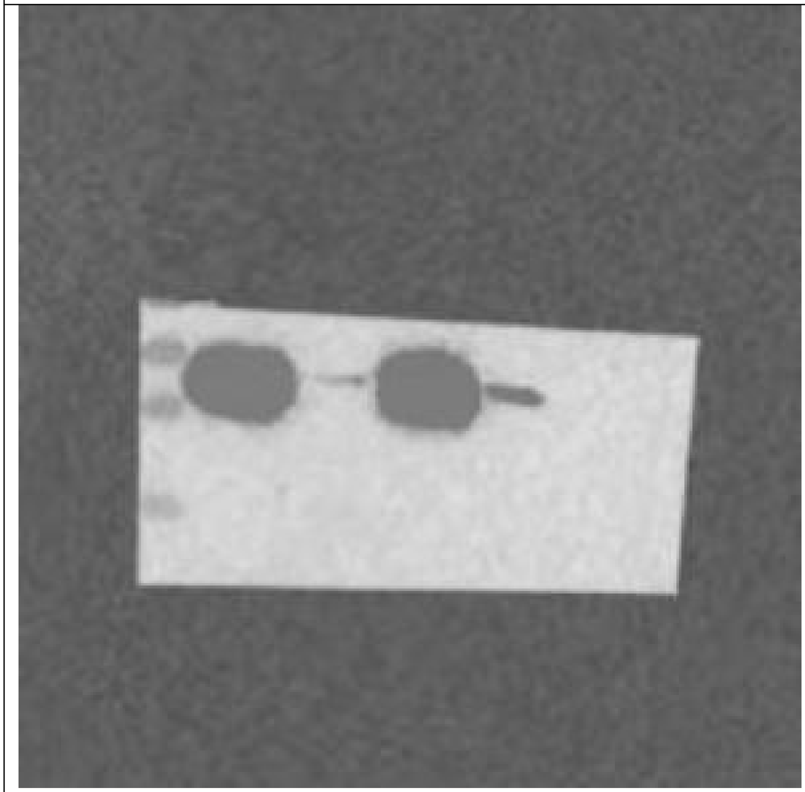

Sample name:

Protein samples of 293T-ACE2 cells transfected with FHL2-his eukaryotic expression plasmid

Target protein:

α -tubulin

MW:

50 KD

Lane: 1,2

Left to Right

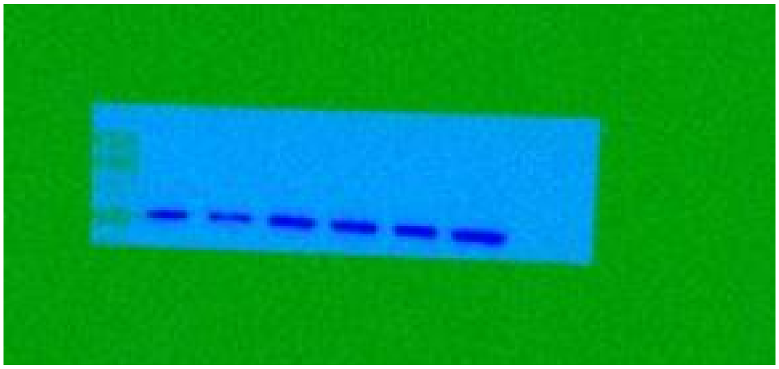

FigS9:Original Western images used for preparing Figure.3C

Sample name:

Protein samples of 0.01MOI WT strain were infected in FHL2 overexpressing Caco2 cells for 12 hours

Target protein:

N

MW:

55 KD

Lane: 1,2

Left to Right

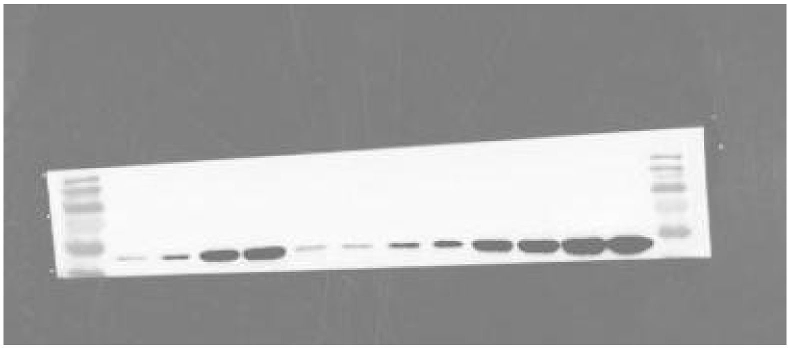

Sample name:

Protein samples of 0.01MOI WT strain were infected in FHL2 overexpressing Caco2 cells for 24 hours

Target protein:

N

MW:

55 KD

Lane: 3,4

Left to Right

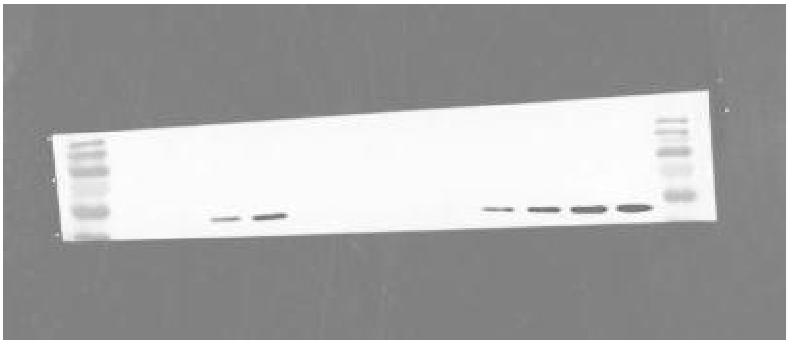

**Sample name:**<br>Protein samples of<br>0.01MOI WT strain were<br>infected in FHL2<br>overexpressing Caco2 cells<br>for 12 hours<br>**Target protein:**<br>GAPDH<br>**MW:**<br>37 KD<br><br>Lane: 1,2<br>Left to Right	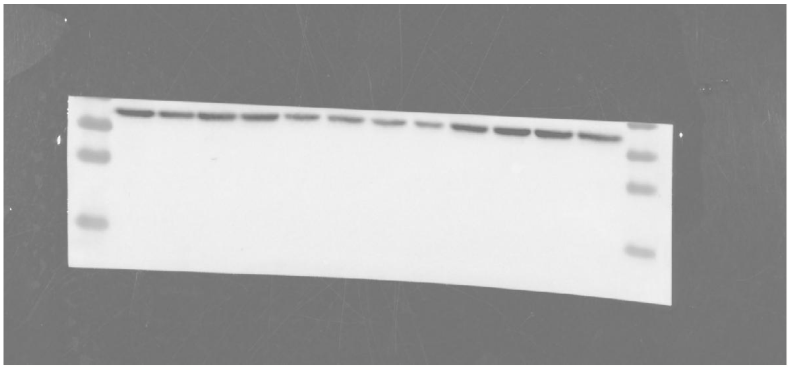
**Sample name:**<br>Protein samples of<br>0.01MOI WT strain were<br>infected in FHL2<br>overexpressing Caco2 cells<br>for 24 hours<br>**Target protein:**<br>GAPDH<br>**MW:**<br>37 KD<br><br>Lane: 3,4<br>Left to Right	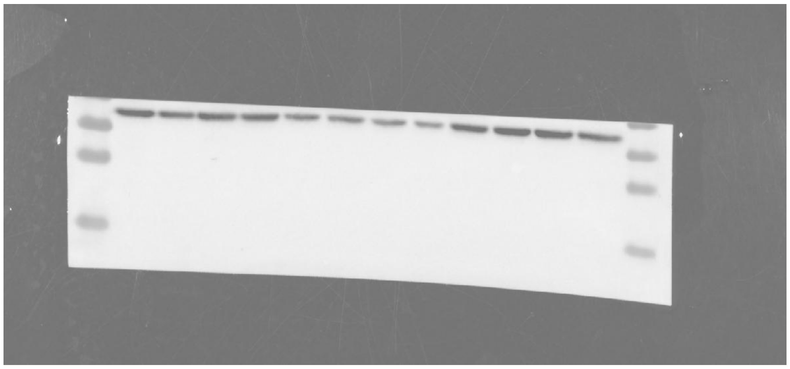

**Sample name:**<br>Protein samples of 0.1MOI WT strain were infected in FHL2 overexpressing Caco2 cells for 12 hours<br>**Target protein:**<br>N<br>**MW:**<br>55 KD<br><br>Lane: 3,4<br>Right to Left	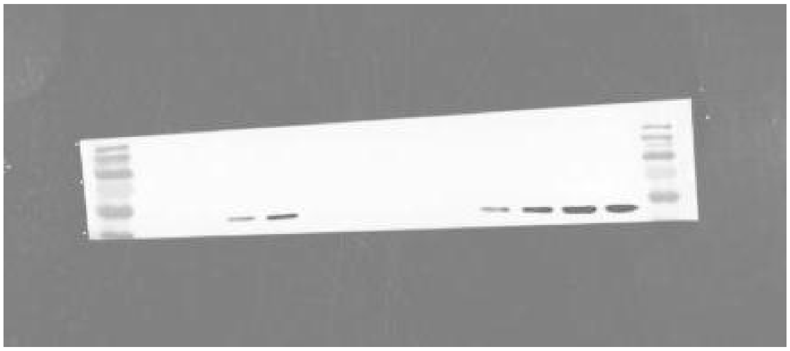
**Sample name:**<br>Protein samples of 0.1MOI WT strain were infected in FHL2 overexpressing Caco2 cells for 24 hours<br>**Target protein:**<br>N<br>**MW:**<br>55 KD<br><br>Lane: 1,2<br>Right to Left	

Sample name:

Protein samples of 0.1MOI WT strain were infected in FHL2 overexpressing Caco2 cells for 12 hours

Target protein:

GAPDH

MW:

37 KD

Lane: 3,4

Right to Left

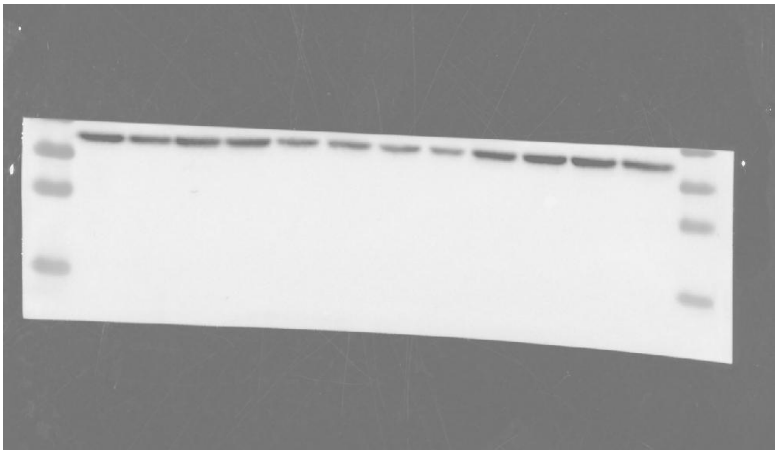

Sample name:

Protein samples of 0.1MOI WT strain were infected in FHL2 overexpressing Caco2 cells for 24 hours

Target protein:

GAPDH

MW:

37 KD

Lane: 1,2

Right to Left

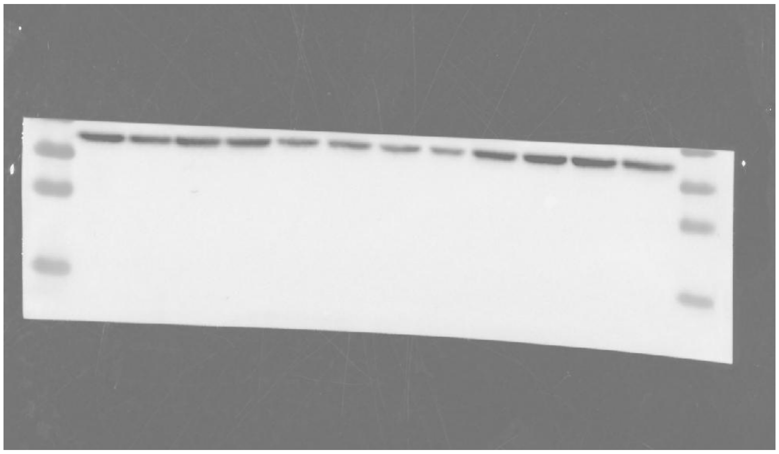

Sample name:

Protein samples of 0.01MOI BA.1 strain were infected in FHL2 overexpressing Caco2 cells for 12 hours

Target protein:

N

MW:

55 KD

Lane: 7,8

Right to Left

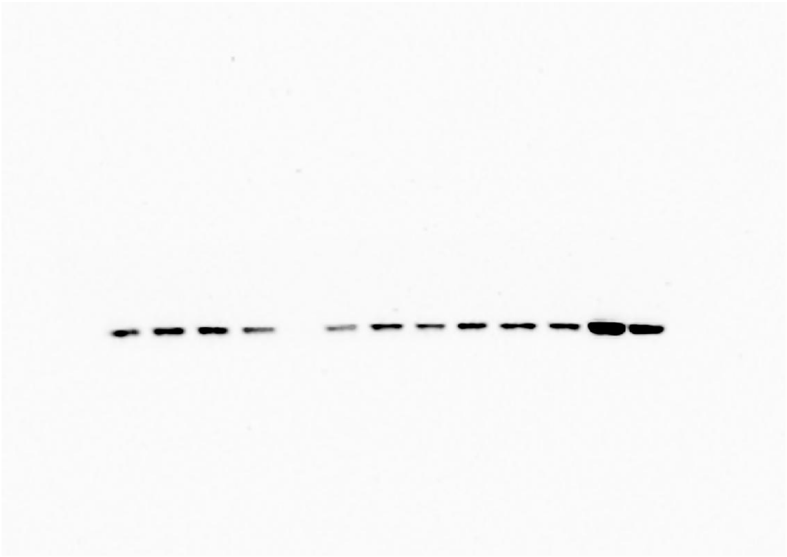

Sample name:

Protein samples of 0.01MOI BA.1 strain were infected in FHL2 overexpressing Caco2 cells for 24 hours

Target protein:

N

MW:

55 KD

Lane: 5,6

Right to Left

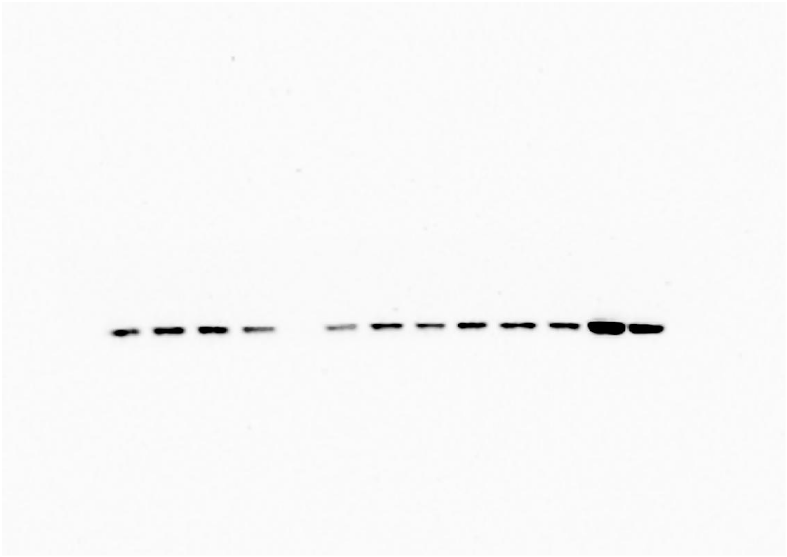

**Sample name:**<br>Protein samples of<br>0.01MOI BA.1 strain were<br>infected in FHL2<br>overexpressing Caco2 cells<br>for 12 hours<br>**Target protein:**<br>GAPDH<br>**MW:**<br>37 KD<br><br>Lane: 7,8<br>Right to Left	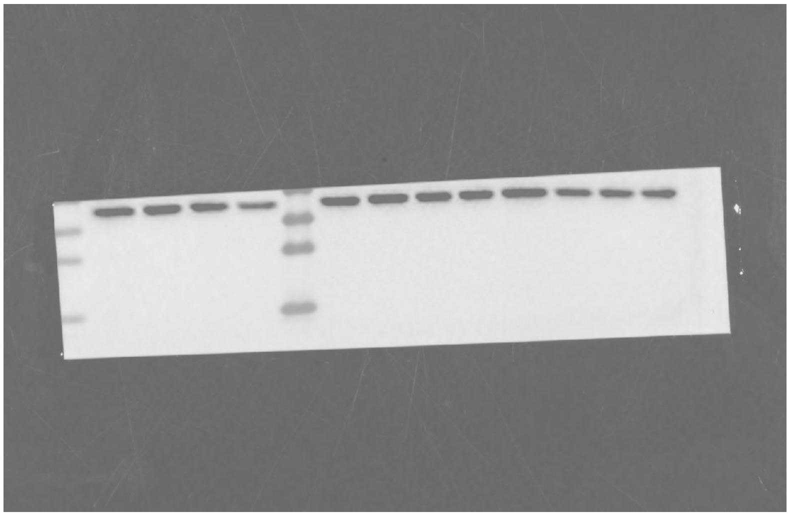
**Sample name:**<br>Protein samples of<br>0.01MOI BA.1 strain were<br>infected in FHL2<br>overexpressing Caco2 cells<br>for 24 hours<br>**Target protein:**<br>GAPDH<br>**MW:**<br>37 KD<br><br>Lane: 5,6<br>Right to Left	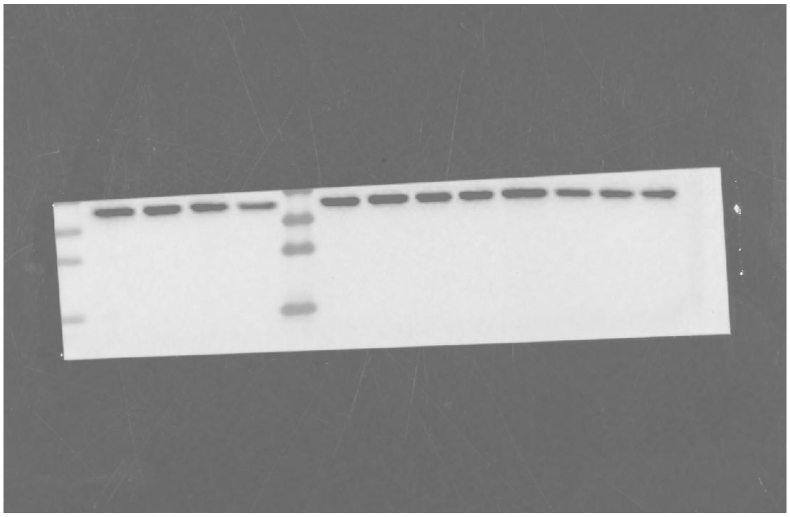

FigS10:Original Western images used for preparing Figure.3D

**Sample name:**<br>Protein samples of 0.01MOI WT strain were infected in FHL2 overexpressing 293T-ACE2 cells for 12 hours<br>**Target protein:**<br>N<br>**MW:**<br>55 KD<br><br>Lane: 1,2<br>Right to Left	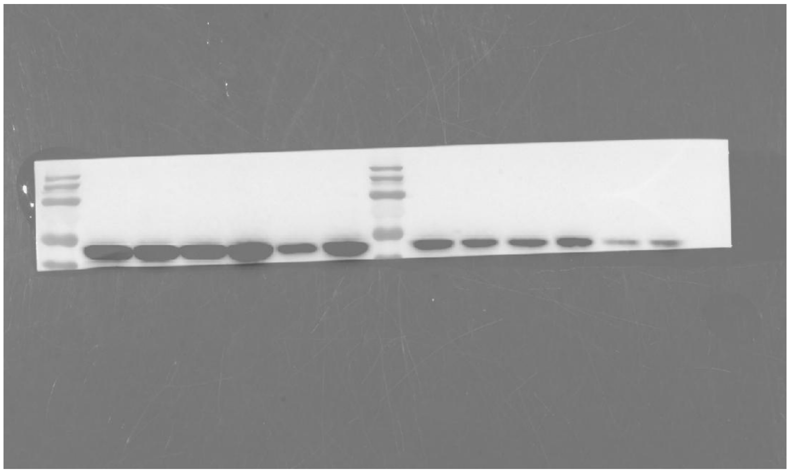
**Sample name:**<br>Protein samples of 0.01MOI WT strain were infected in FHL2 overexpressing 293T-ACE2 cells for 24 hours<br>**Target protein:**<br>N<br>**MW:**<br>55 KD<br><br>Lane: 3,4<br>Right to Left	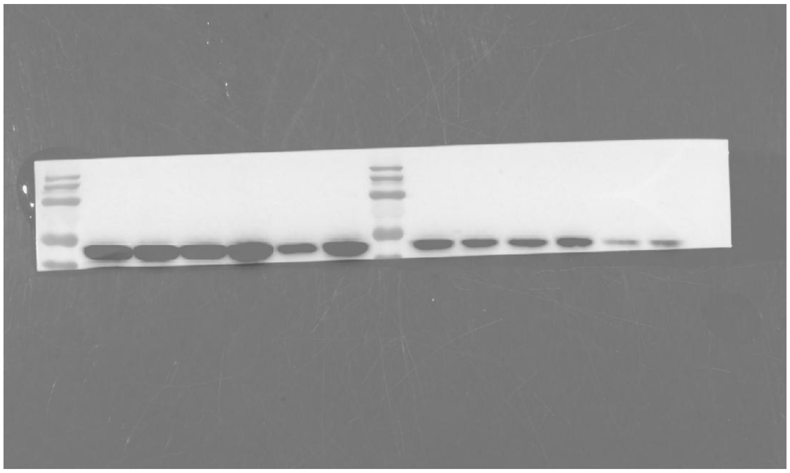

Sample name:

Protein samples of 0.01MOI WT strain were infected in FHL2 overexpressing 293T-ACE2 cells for 12 hours

Target protein:

GAPDH

MW:

37 KD

Lane: 1,2

Right to Left

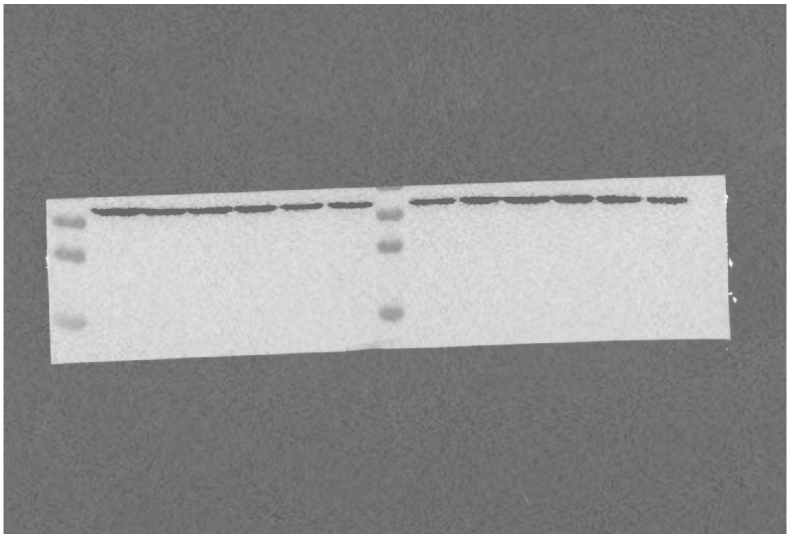

Sample name:

Protein samples of 0.01MOI WT strain were infected in FHL2 overexpressing 293T-ACE2 cells for 24 hours

Target protein:

GAPDH

MW:

37 KD

Lane: 3,4

Right to Left

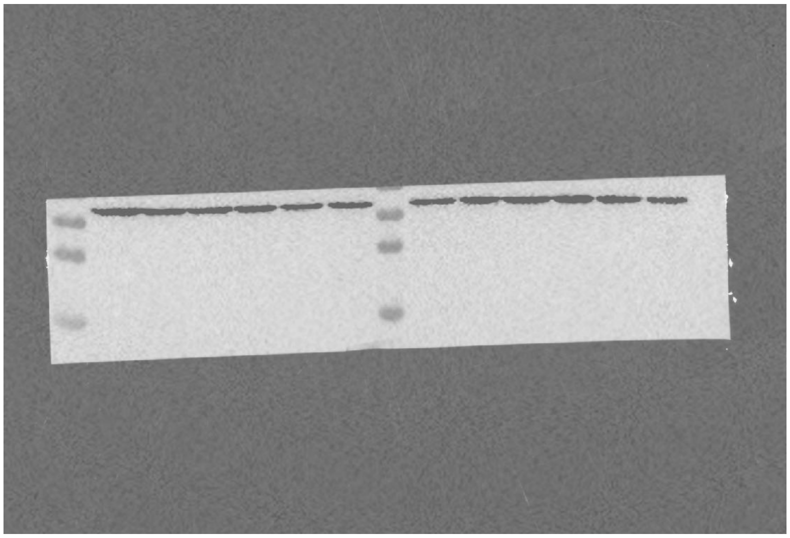

Sample name:

Protein samples of 0.01MOI BA.1 strain were infected in FHL2 overexpressing 293T-ACE2 cells for 12 hours

Target protein:

N

MW:

55 KD

Lane: 5,6

Left to Right

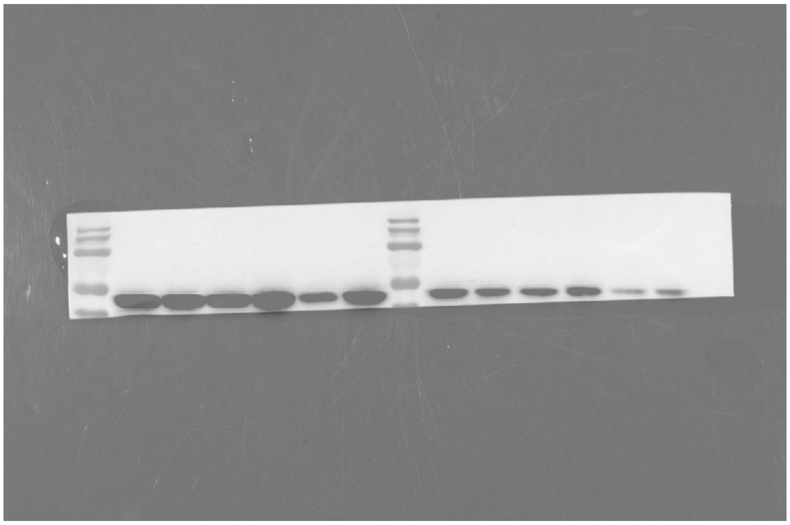

Sample name:

Protein samples of 0.01MOI BA.1 strain were infected in FHL2 overexpressing 293T-ACE2 cells for 24 hours

Target protein:

N

MW:

55 KD

Lane: 3,4

Left to Right

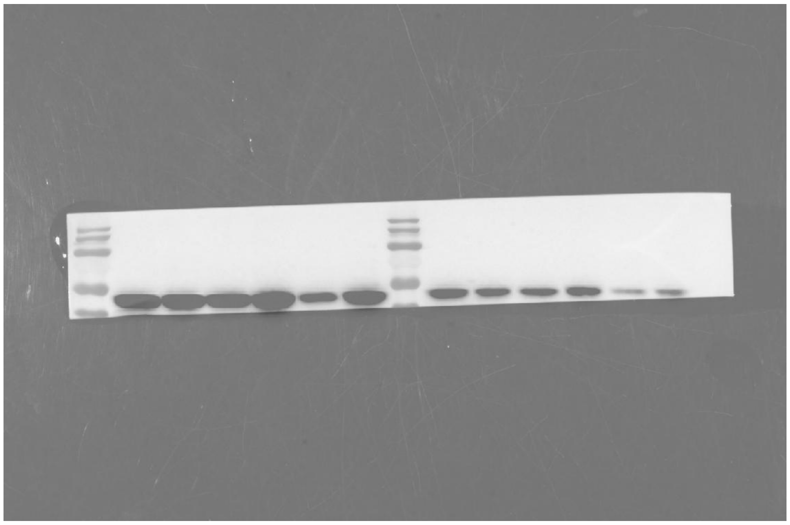

**Sample name:**<br>Protein samples of 0.01MOI BA.1 strain were infected in FHL2 overexpressing 293T-ACE2 cells for 12 hours<br>**Target protein:**<br>GAPDH<br>**MW:**<br>37 KD<br><br>Lane: 5,6<br>Left to Right	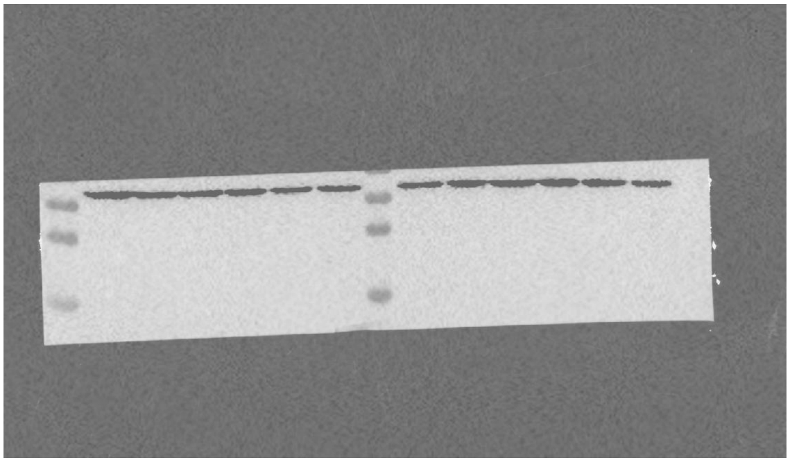
**Sample name:**<br>Protein samples of 0.01MOI BA.1 strain were infected in FHL2 overexpressing 293T-ACE2 cells for 24 hours<br>**Target protein:**<br>GAPDH<br>**MW:**<br>37 KD<br><br>Lane: 3,4<br>Left to Right	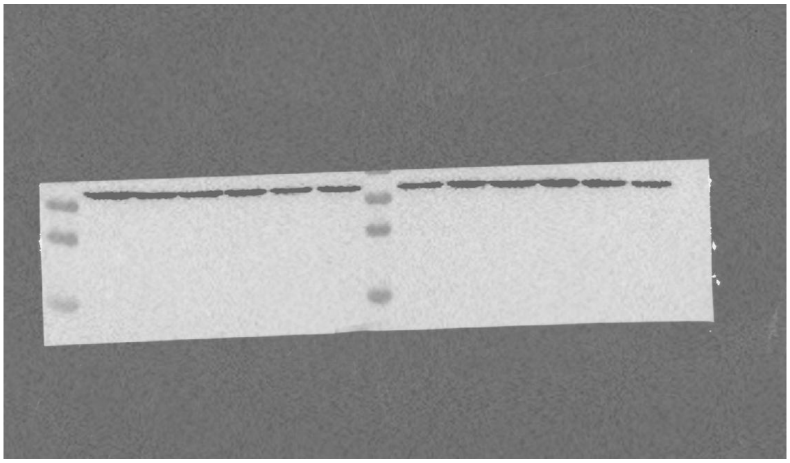

FigS11:Original Western images used for preparing Figure.3E

**Sample name:**<br>Protein samples of Caco2 cells transfected with FHL2-his eukaryotic expression plasmid (2 μ g、1 μ g、0.5 μ g、0 μ g)<br>**Target protein:**<br>FHL2-his<br>**MW:**<br>32 KD<br><br>Lane: 1,2,3,4<br>Left to Right	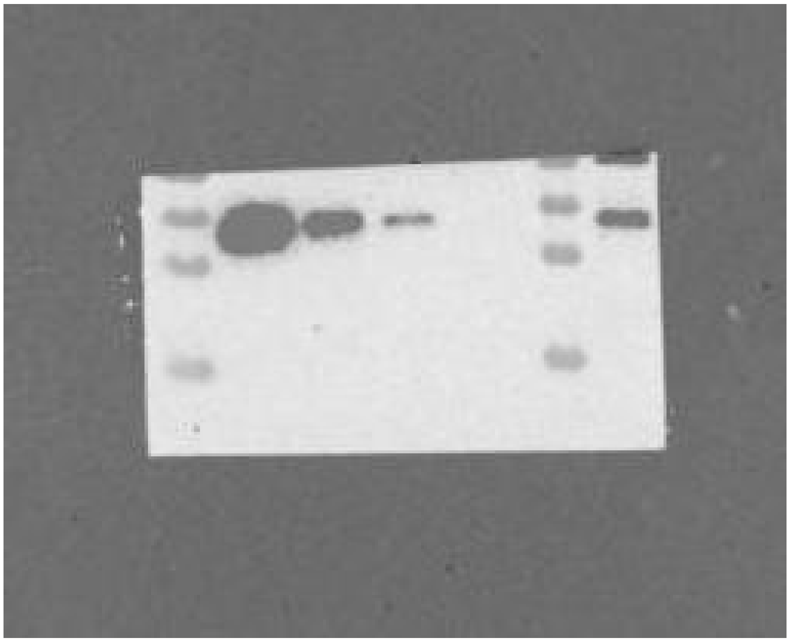
**Sample name:**<br>Protein samples of Caco2 cells transfected with FHL2-his eukaryotic expression plasmid (2 μ g、1 μ g、0.5 μ g、0 μ g)<br>**Target protein:**<br>α -tubulin<br>**MW:**<br>50 KD<br><br>Lane: 1,2,3,4<br>Left to Right	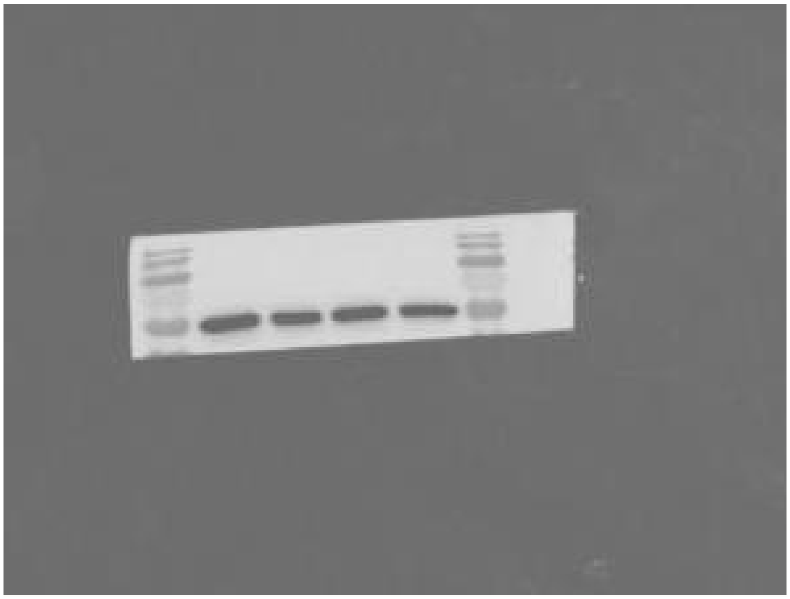

Sample name:

Protein samples of 0.01MOI WT strain were infected in FHL2 overexpressing 293T-ACE2 cells for 12 hours

Target protein:

N

MW:

55 KD

Lane: 3,4,5,6

Left to Right

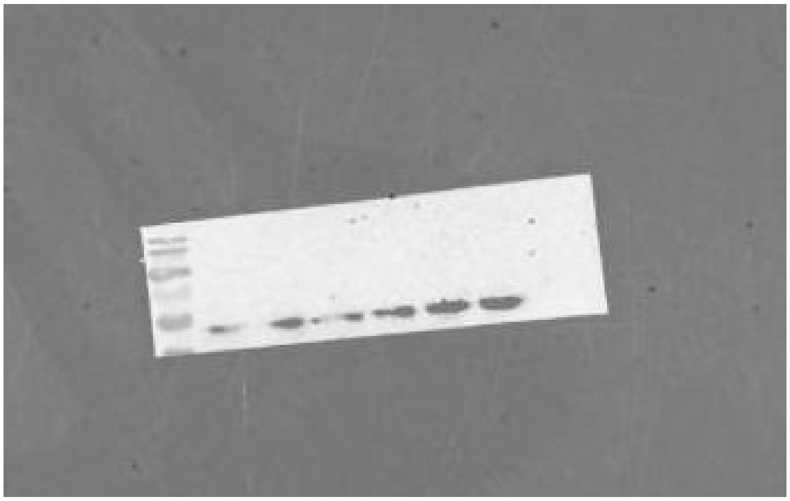

Sample name:

Protein samples of 0.1MOI WT strain were infected in FHL2 overexpressing Caco2 cells for 12 hours

Target protein:

GAPDH

MW:

37 KD

Lane: 3,4,5,6

Left to Right

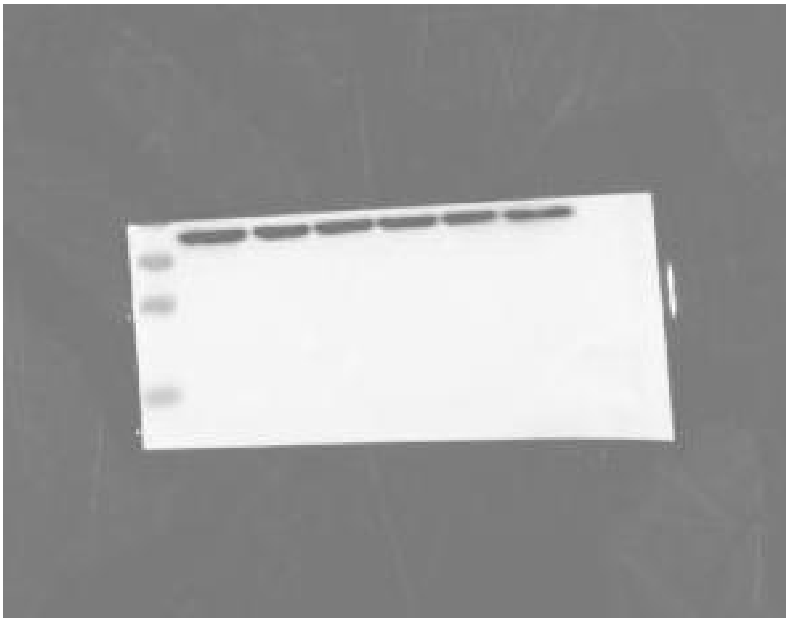

FigS12:Original Western images used for preparing Figure.5A

Sample name:

Protein samples of transfected with shRNA in Caco2 cells

Target protein:

IRF-3

MW:

55 KD

Lane: 3,4

Left to Right

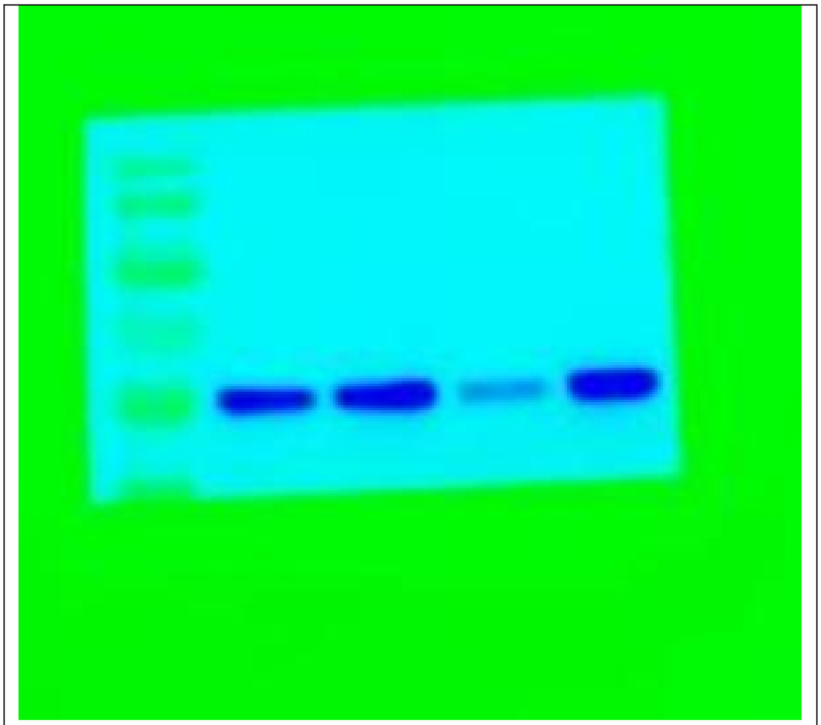

Sample name:

Protein samples of transfected with shRNA in Caco2 cells

Target protein:

GAPDH

MW:

37 KD

Lane: 3,4

Left to Right

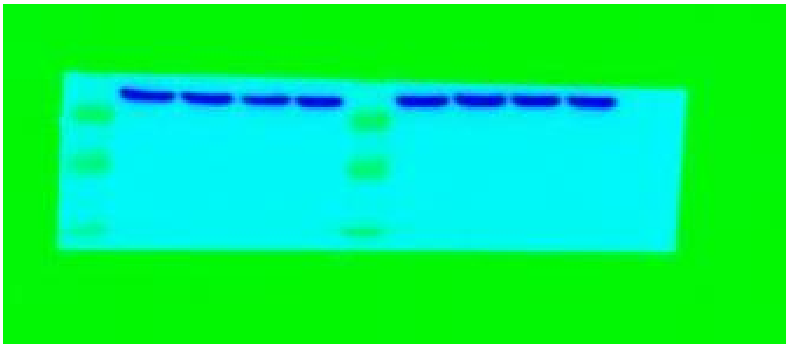

FigS13:Original Western images used for preparing Figure.5C

**Sample name:**<br>Protein samples of Caco2 cells transfected with FHL2-his eukaryotic expression plasmid<br>**Target protein:**<br>IRF-3<br>**MW:**<br>55 KD<br><br>Lane: 3,4<br>Left to Right	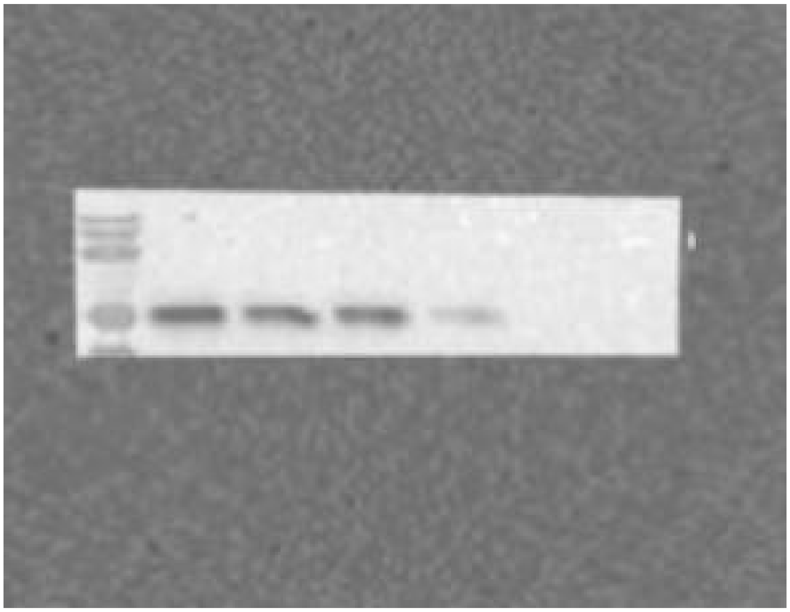
**Sample name:**<br>Protein samples of Caco2 cells transfected with FHL2-his eukaryotic expression plasmid<br>**Target protein:**<br>GAPDH<br>**MW:**<br>37 KD<br><br>Lane: 7,8<br>Left to Right	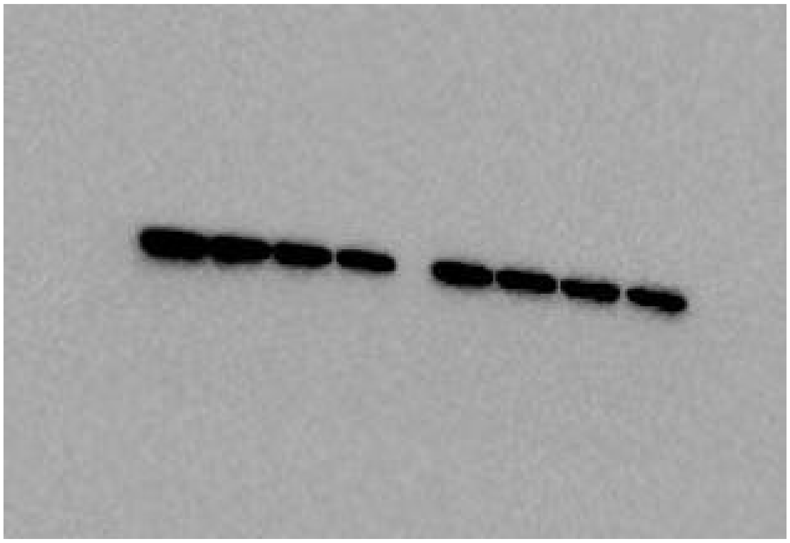

FigS14:Original Western images used for preparing Figure.5E

**Sample name:**<br>Protein samples of FHL2 overexpressing Caco2 cells stimulated by RNA (Nuclei)<br>**Target protein:**<br>IRF-3<br>**MW:**<br>55 KD<br><br>Lane: 9,10,11,12<br>Left to Right	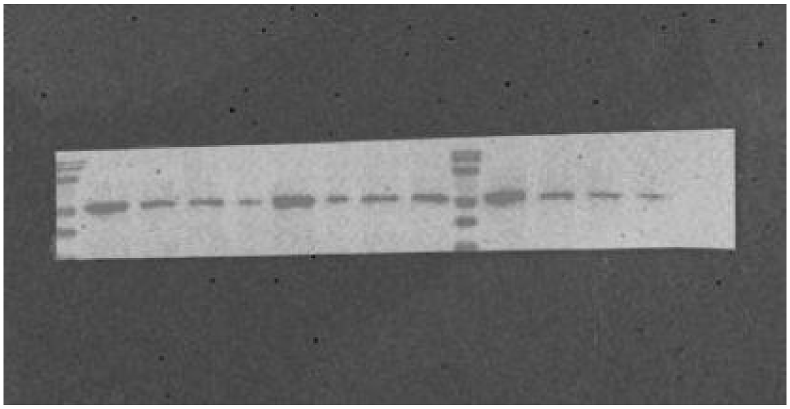
**Sample name:**<br>Protein samples of FHL2 overexpressing Caco2 cells stimulated by RNA (Nuclei)<br>**Target protein:**<br>Lamin A/C<br><br>Second row<br>Lane: 9,10,11,12<br>Left to Right	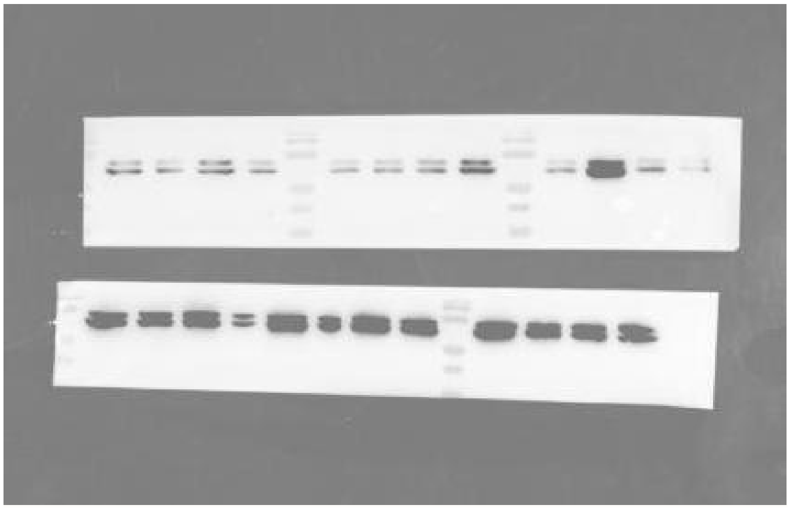

Sample name:

Protein samples of FHL2 overexpressing Caco2 cells stimulated by RNA (Cyto)

Target protein:

IRF-3

MW:

55 KD

Lane: 9,10,11,12

Left to Right

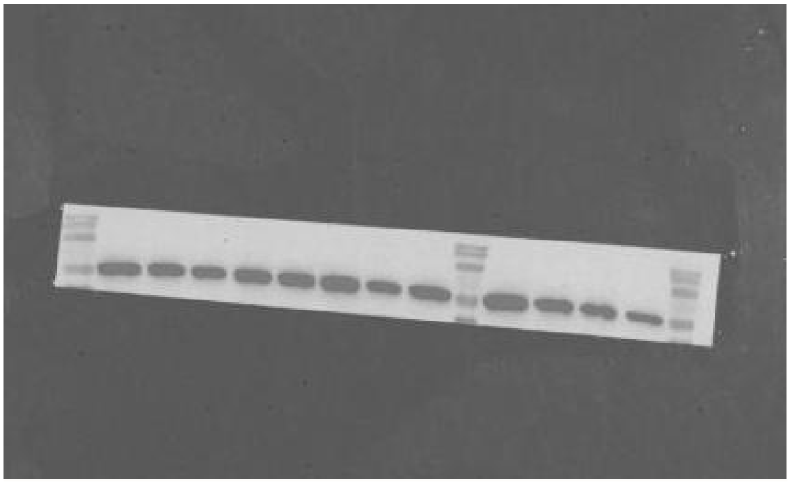

Sample name:

Protein samples of FHL2 overexpressing Caco2 cells stimulated by RNA (Cyto)

Target protein:

GAPDH

MW:

37 KD

Lane: 9,10,11,12

Left to Right

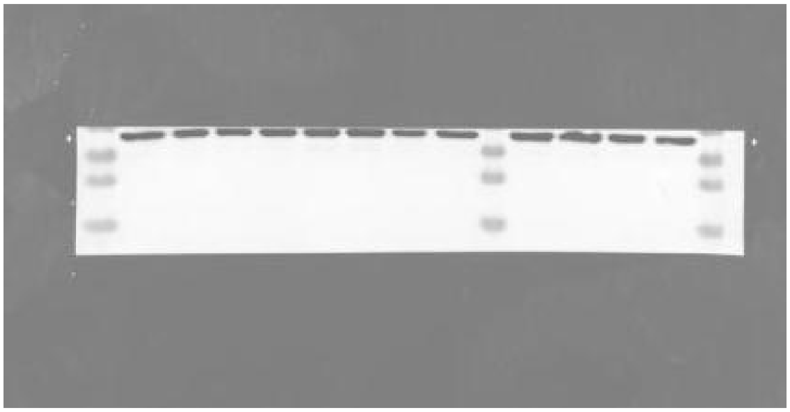

Sample name:

Protein samples of FHL2 overexpressing Caco2 cells stimulated by RNA (Total)

Target protein:

IRF-3

MW:

55 KD

Lane: 5,6,7,8

Left to Right

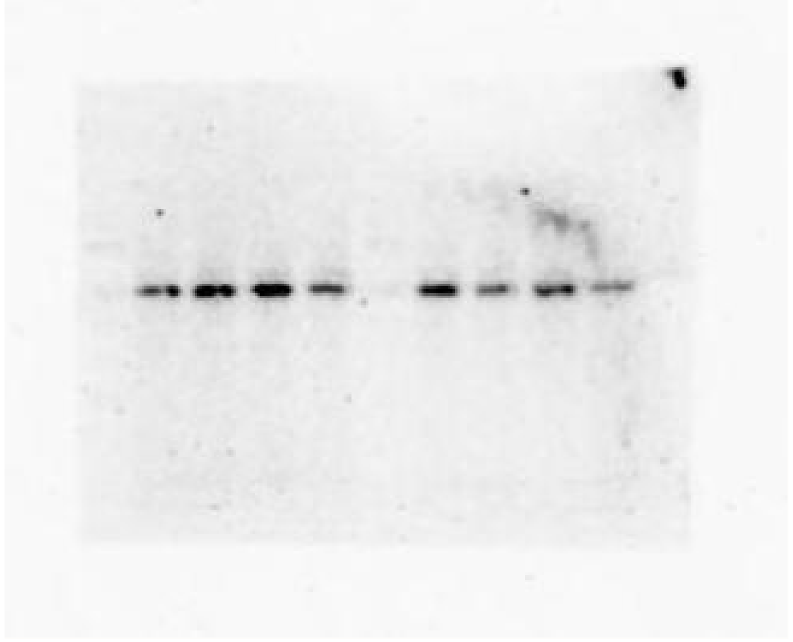

Sample name:

Protein samples of FHL2 overexpressing Caco2 cells stimulated by RNA (Total)

Target protein:

GAPDH

MW:

37 KD

Lane: 5,6,7,8

Left to Right

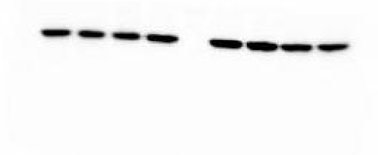

FigS15:Original Western images used for preparing Figure.5F

**Sample name:**<br>Protein samples of FHL2 overexpressing Caco2 cells stimulated by virus<br>**Target protein:**<br>P-IRF-3<br>**MW:**<br>55 KD<br><br>Lane: 3,4,5,6<br>Right to Left	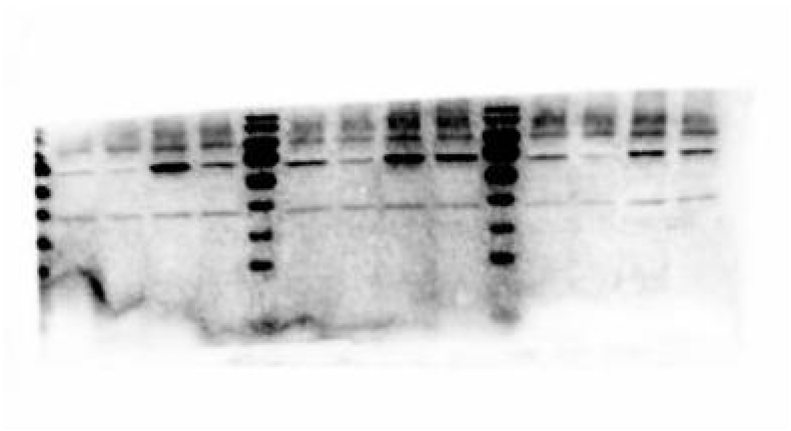
**Sample name:**<br>Protein samples of FHL2 overexpressing Caco2 cells stimulated by virus<br>**Target protein:**<br>IRF-3<br>**MW:**<br>55 KD<br><br>Lane: 3,4,5,6<br>Right to Left	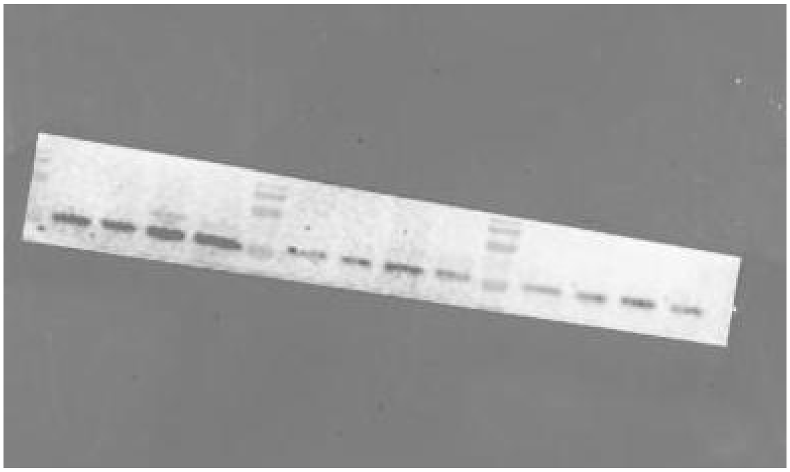

Sample name:

Protein samples of FHL2 overexpressing Caco2 cells stimulated by virus

Target protein:

GAPDH

MW:

37 KD

Lane: 3,4,5,6

Right to Left

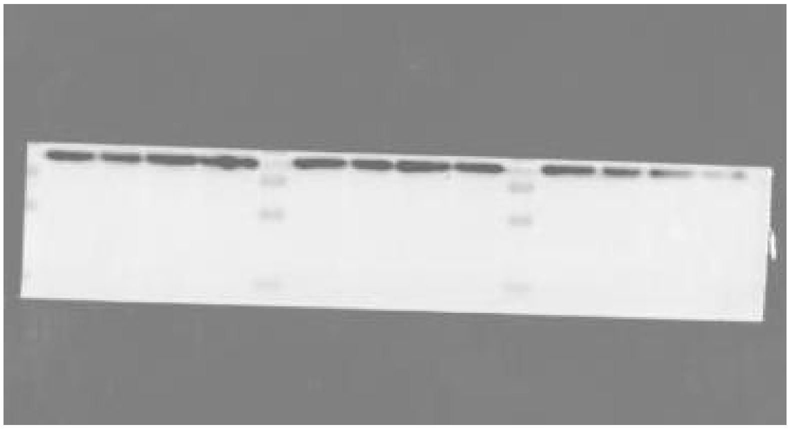

FigS16:Original Western images used for preparing Figure.5F

Sample name:

Protein samples of FHL2 knockdown Caco2 cells stimulated by virus

Target protein:

P-IRF-3

MW:

55 KD

Lane: 1,2,3,4

Left to Right

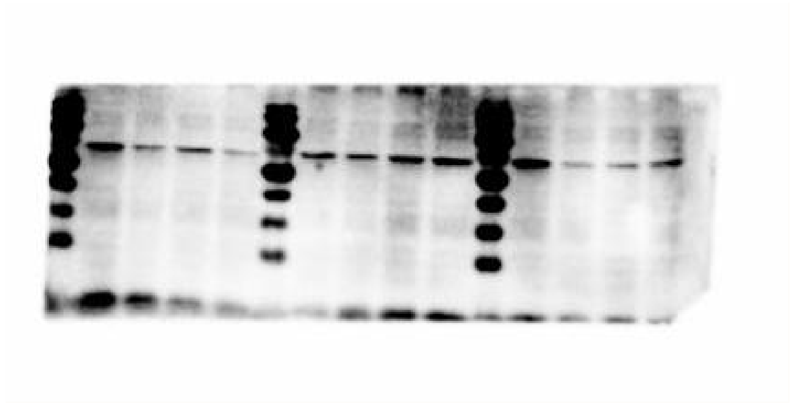

Sample name:

Protein samples of FHL2 knockdown Caco2 cells stimulated by virus

Target protein:

IRF-3

MW:

55 KD

Lane: 1,2,3,4

Left to Right

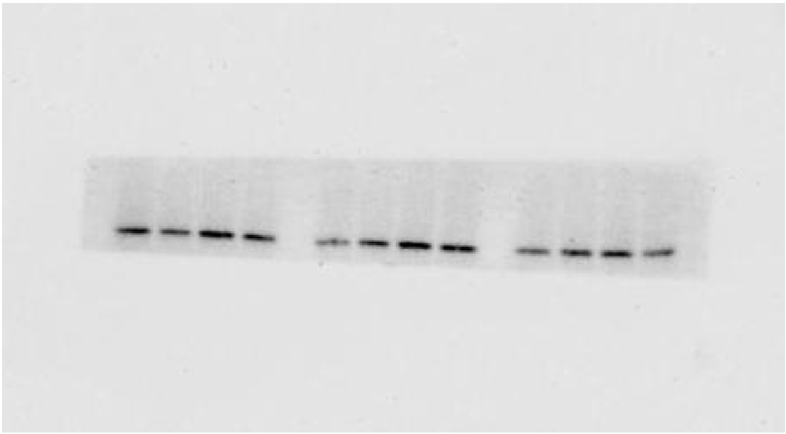

Sample name:

Protein samples of FHL2 knockdown Caco2 cells stimulated by virus

Target protein:

GAPDH

MW:

37 KD

Lane: 1,2,3,4

Left to Right

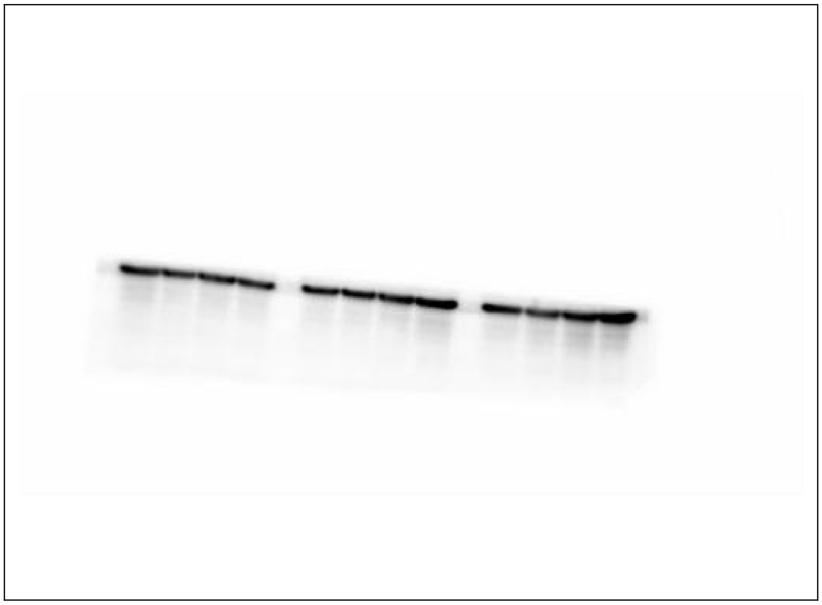

Supplement: Supplementary file 1 [file ijms-25-00353-s001.zip › RAW Images for WB.pdf]
